# Supplementary material for: A multi-omic dissection of super-enhancer driven oncogenic gene expression programs in ovarian cancer
Source: Nat Commun. 2022 Jul 22;13:4247. doi: 10.1038/s41467-022-31919-8 (PMC9307778; doi:10.1038/s41467-022-31919-8)
Supplement: Supplementary file 1 — Supplementary Information [file 41467_2022_31919_MOESM1_ESM.pdf]

A multi-omic dissection of super-enhancer driven oncogenic gene expression programs in ovarian cancer

Michael R. Kelly[1,2,6], Kamila Wisniewska[1,6], Matthew J. Regner[1,2], Michael W. Lewis[1], Andrea A. Perreault[3], Eric S. Davis[2], Douglas H. Phanstiel[1,3,4], Joel S. Parker[1,2,5] and Hector L. Franco[1,2,5,*]

1. The Lineberger Comprehensive Cancer Center, University of North Carolina at Chapel Hill, Chapel Hill, NC, 27599, USA
2. Bioinformatics and Computational Biology Graduate Program, University of North Carolina at Chapel Hill, Chapel Hill, NC, 27599, USA
3. Thurston Arthritis Research Center, University of North Carolina at Chapel Hill, Chapel Hill, NC, 27599, USA
4. Department of Cell Biology & Physiology, University of North Carolina at Chapel Hill, Chapel Hill, NC, 27599, USA
5. The Department of Genetics, School of Medicine, University of North Carolina at Chapel Hill, Chapel Hill, NC, 27599, USA
6. These authors contributed equally.

* Correspondence: hfranco@med.unc.edu

This PDF file contains the following Supplementary Information:

Supplementary Figures 1-12

Supplementary Tables 1-2

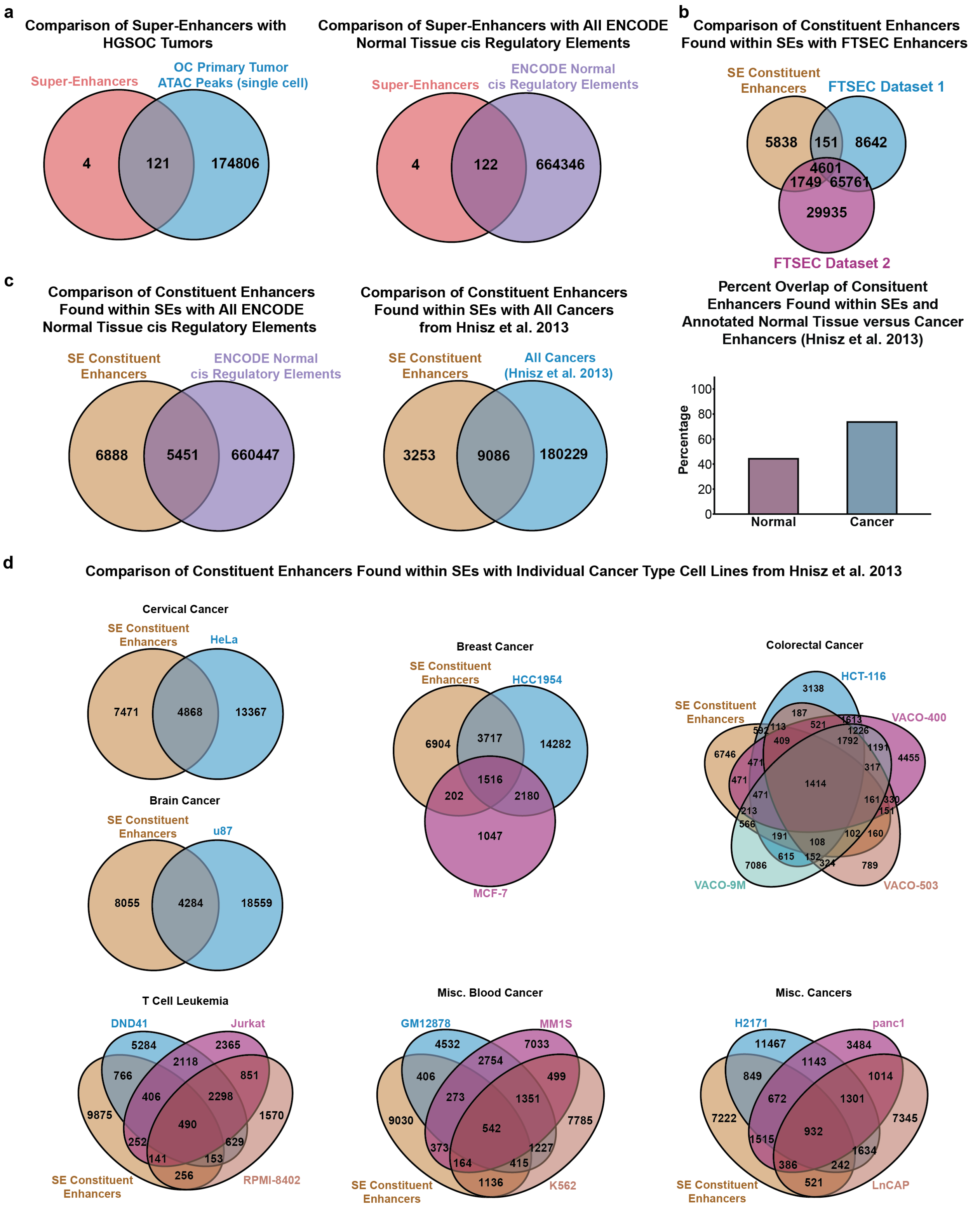
a
Comparison of Super-Enhancers with HGSOC Tumors
Super-Enhancers
OC Primary Tumor ATAC Peaks (single cell)
4
121
174806
Comparison of Super-Enhancers with All ENCODE Normal Tissue cis Regulatory Elements
Super-Enhancers
ENCODE Normal cis Regulatory Elements
4
122
664346
b
Comparison of Constituent Enhancers Found within SEs with FTSEC Enhancers
SE Constituent Enhancers
FTSEC Dataset 1
5838
151
8642
4601
1749
65761
29935
FTSEC Dataset 2
c
Comparison of Constituent Enhancers Found within SEs with All ENCODE Normal Tissue cis Regulatory Elements
SE Constituent Enhancers
ENCODE Normal cis Regulatory Elements
6888
5451
660447
Comparison of Constituent Enhancers Found within SEs with All Cancers from Hnisz et al. 2013
SE Constituent Enhancers
All Cancers (Hnisz et al. 2013)
3253
9086
180229
Percent Overlap of Consituent Enhancers Found within SEs and Annotated Normal Tissue versus Cancer Enhancers (Hnisz et al. 2013)
Percentage
100
80
60
40
20
0
Normal
Cancer
d
Comparison of Constituent Enhancers Found within SEs with Individual Cancer Type Cell Lines from Hnisz et al. 2013
Cervical Cancer
SE Constituent Enhancers
HeLa
7471
4868
13367
Brain Cancer
SE Constituent Enhancers
u87
8055
4284
18559
Breast Cancer
SE Constituent Enhancers
HCC1954
6904
3717
14282
1516
202
2180
1047
MCF-7
Colorectal Cancer
HCT-116
SE Constituent Enhancers
VACO-400
VACO-9M
VACO-503
3138
187
521
613
1226
592
113
409
1792
1191
6746
471
317
4455
471
1414
471
161
330
213
151
566
191
108
102
160
615
152
7086
324
789
T Cell Leukemia
DND41
Jurkat
5284
2365
766
2118
851
406
2298
9875
490
1570
252
629
141
153
256
SE Constituent Enhancers
RPMI-8402
Misc. Blood Cancer
GM12878
MM1S
4532
7033
406
2754
499
273
1351
9030
542
7785
373
1227
164
415
1136
SE Constituent Enhancers
K562
Misc. Cancers
H2171
panc1
11467
3484
849
1143
1014
672
1301
7222
932
7345
1515
1634
386
242
521
SE Constituent Enhancers
LnCAP

Supplementary Figure 1. OVCAR3 Super-Enhancers Show Enrichment of Activity in High Grade Serous Ovarian Cancer Patient Tumors and Cancerous Tissues

- **a.** Overlap of hg38 detected super-enhancers (SEs) (lifted-over from Figure 1) with enhancers detected in the cancer cell fraction of high grade serous ovarian cancer (HGSOC) tumors from Regner et al.[1] (*left*). Overlap of hg19 detected SEs (from Figure 1) with all enhancer elements contained in the ENCODE database (*right*).
- **b.** Overlap of constituent enhancers found within SEs (Figure 1) and normal fallopian tube secretory epithelial cell (FTSEC) enhancer regions documented in Coetzee et al. 2015[2].
- **c.** Left: Overlap of constituent enhancers found within SEs (Figure 1) and all normal tissue *cis* regulatory elements in ENCODE. Middle: All cancer associated enhancers documented in the Hnisz et al. 2013 research article[3]. Right: Breakdown of the percentage of the constituent enhancers found in normal cells (purple) vs cancer cells (blue).
- **d.** Overlap of constituent enhancers found within SEs (Figure 1) and all cancer associated enhancers documented in the Hnisz et al. 2013 research article[3] broken down by cancer type.

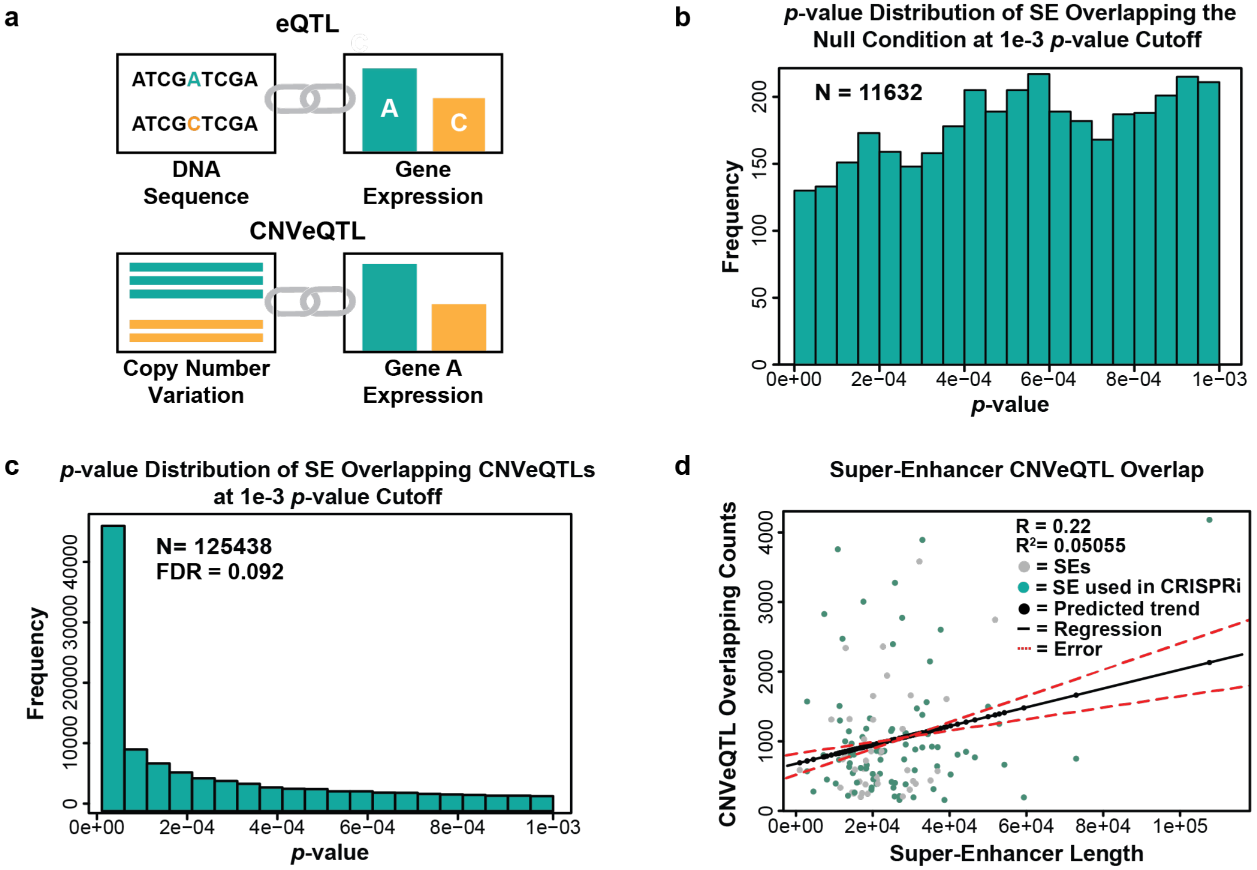

Supplementary Figure 2: Copy Number Expression Quantitative Loci Analysis (CNVeQTL) in Ovarian Cancer Patients Nominates Salient Super-Enhancers.

- **a.** Cartoon comparing expression-based quantitative trait loci (eQTL) analysis versus copy-number-based expression quantitative trait loci (CNVeQTL) analysis.
- **b.** Histogram of CNVeQTL p-values from one of the 100k permuted null distributions used to calculate the empirically derived false discovery rate (eFDR) of the super-enhancer (SE) overlapping CNVeQTLs. Median number of CNVeQTLs at p-value threshold of $1x10^{-3}$ across all 100k null distributions is shown (n = 11,632).
- **c.** Histogram of CNVeQTL p-values from the experimental distribution (SE overlapping CNVeQTLs). The eFDR was determined by comparing the amount of significant CNVeQTL in the experimental group versus the median CNVeQTL count of the 100k permuted nulls.
- **d.** Scatter plot showing the relationship between SE length (in base pairs) and the number of significantly associated CNVeQTLs. The direct association between genomic size and CNVeQTL count shown is by the regression line in black with error bounds depicted as dashed red lines. Dots that represent the SEs that were included into the CRISPRi screen are colored in teal.

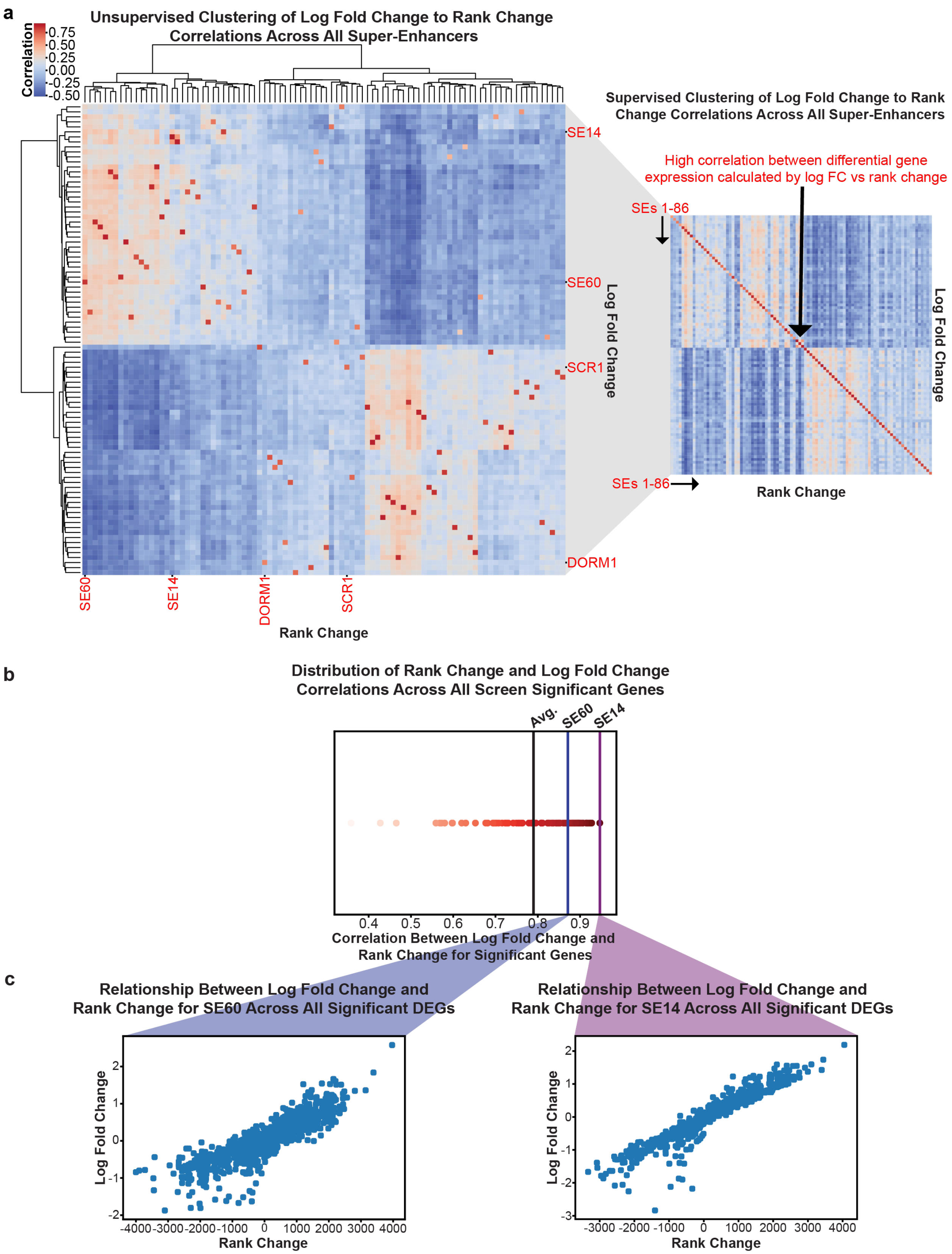

a
Unsupervised Clustering of Log Fold Change to Rank Change Correlations Across All Super-Enhancers
Correlation
0.75
0.50
0.25
0.00
-0.25
-0.50
SE14
SE60
SCR1
DORM1
Log Fold Change
SE60
SE14
DORM1
SCR1
Rank Change
Supervised Clustering of Log Fold Change to Rank Change Correlations Across All Super-Enhancers
High correlation between differential gene expression calculated by log FC vs rank change
SEs 1-86
SEs 1-86
Log Fold Change
Rank Change
b
Distribution of Rank Change and Log Fold Change Correlations Across All Screen Significant Genes
Avg.
SE60
SE14
0.4
0.5
0.6
0.7
0.8
0.9
Correlation Between Log Fold Change and Rank Change for Significant Genes
c
Relationship Between Log Fold Change and Rank Change for SE60 Across All Significant DEGs
Log Fold Change
2
1
0
-1
-2
-4000
-3000
-2000
-1000
0
1000
2000
3000
4000
Rank Change
Relationship Between Log Fold Change and Rank Change for SE14 Across All Significant DEGs
Log Fold Change
2
1
0
-1
-2
-3
-3000
-2000
-1000
0
1000
2000
3000
4000
Rank Change

Supplementary Figure 3: Expression Analysis of the CRISPRi Screen Reveals High Correlation with Rank Change Values

- **a.** Unsupervised hierarchically clustered heatmap showing the relationship between Log2 Fold Change and Rank Change values for all super-enhancers and their detected target genes. Denoted in red are SE60 and 14 as well as controls (*left*). The same correlation data is provided in a supervised heatmap where the LFC and Rank Change for every SE follow the paired diagonal (*right*).
- **b.** Distribution of all Rank Change to LFC correlations for every SE. The average correlation (between Rank Change and LFC) of all SEs is denoted in black, the correlation between LFC and RC values for SE60 significant DEGs is denoted in blue, and SE14 is denoted in purple.
- **c.** Scatterplot showing the relationship between rank change and log2 fold change for all detected DEGs (from any super-enhancer) genes in SE60 (*left*) and SE14 (*right*).

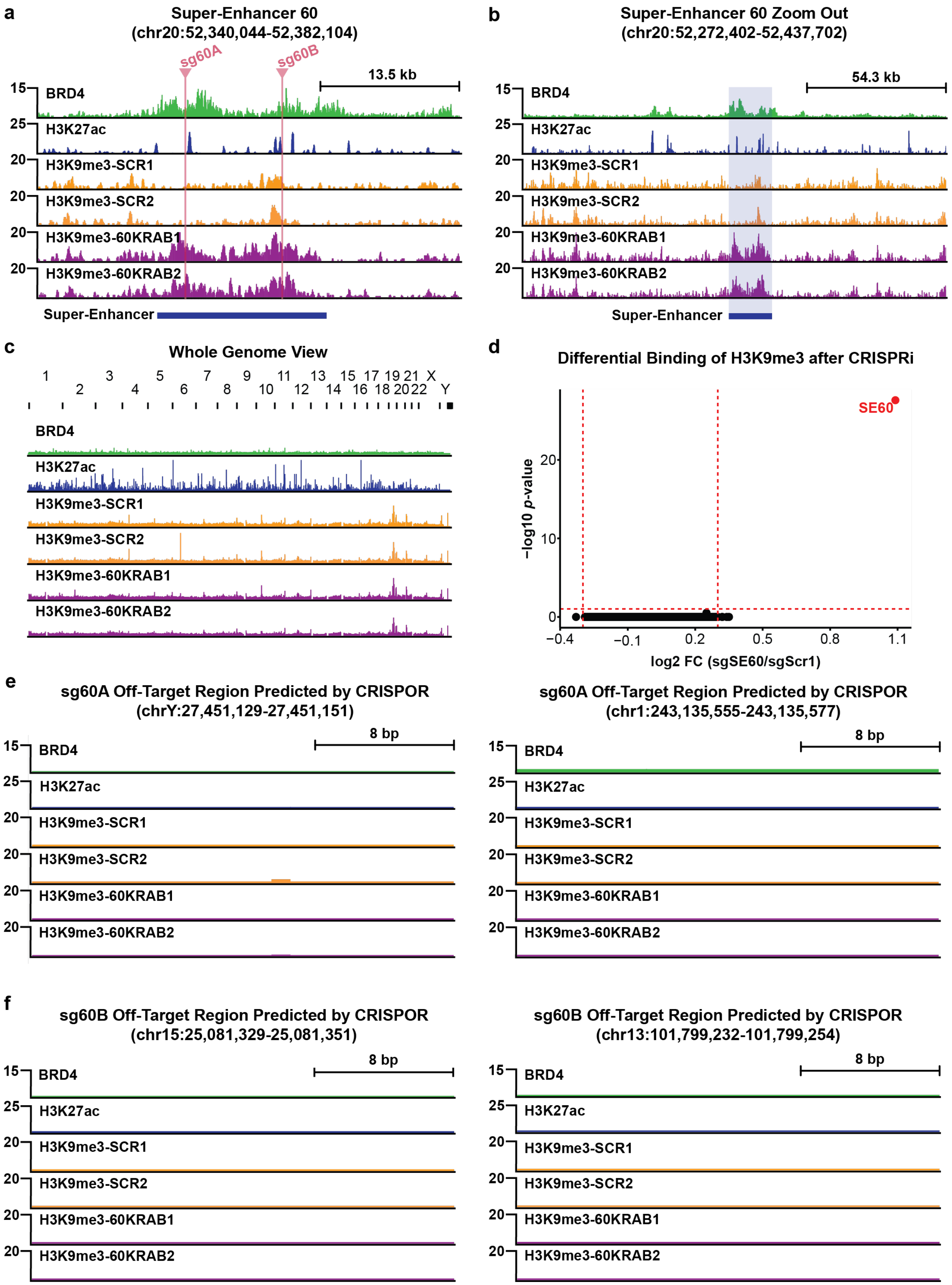

a
Super-Enhancer 60
(chr20:52,340,044-52,382,104)
sg60A
sg60B
13.5 kb
BRD4
H3K27ac
H3K9me3-SCR1
H3K9me3-SCR2
H3K9me3-60KRAB1
H3K9me3-60KRAB2
Super-Enhancer
b
Super-Enhancer 60 Zoom Out
(chr20:52,272,402-52,437,702)
54.3 kb
BRD4
H3K27ac
H3K9me3-SCR1
H3K9me3-SCR2
H3K9me3-60KRAB1
H3K9me3-60KRAB2
Super-Enhancer
c
Whole Genome View
BRD4
H3K27ac
H3K9me3-SCR1
H3K9me3-SCR2
H3K9me3-60KRAB1
H3K9me3-60KRAB2
d
Differential Binding of H3K9me3 after CRISPRi
SE60
−log10 p-value
log2 FC (sgSE60/sgScr1)
e
sg60A Off-Target Region Predicted by CRISPOR
(chrY:27,451,129-27,451,151)
sg60A Off-Target Region Predicted by CRISPOR
(chr1:243,135,555-243,135,577)
8 bp
f
sg60B Off-Target Region Predicted by CRISPOR
(chr15:25,081,329-25,081,351)
sg60B Off-Target Region Predicted by CRISPOR
(chr13:101,799,232-101,799,254)
8 bp

Supplementary Figure 4: H3K9me3 ChIP-Seq Reinforces Targeted CRISPRi of Super-Enhancer 60

- **a.** Browser track view of the SE60 locus investigating the amount of H3K9me3 in scramble negative controls and SE60-KRAB samples. sgRNA target sites are labeled in pink. There is an enrichment in H3K9me3 with the SE60-KRAB condition relative to the scramble controls.
- **b.** Zoomed out browser track view of the SE60 locus investigating the amount of H3K9me3 in scramble controls and SE60-KRAB samples. There is an enrichment in the SE60-KRAB condition relative to the scramble controls that covers ~20kb around the SE60 sgRNA target sites. Blue shadow denotes SE60 location.
- **c.** Browser track of the entire human genome investigating the amount of H3K9me3 in scramble controls and SE60-KRAB samples. There are no clear differences between scramble and SE60-KRAB samples.
- **d.** Volcano plot depicting the results of the differential binding analysis between scramble guide controls and SE60-KRAB targeted knockdown samples, significant regions are denoted in red.
- **e.** Browser track view investigating the amount of H3K9me3 in scramble controls and SE60-KRAB sample for the first (*left*) and second (*right*) highest predicted off-targets of the SE60A sgRNA. There are no clear differences between scramble and SE60-KRAB samples.
- **f.** Browser track view investigating the amount of H3K9me3 in scramble controls and SE60-KRAB samples for the first (*left*) and second (*right*) highest predicted off-targets of the SE60B guide. There are no clear differences between scramble and SE60-KRAB samples.

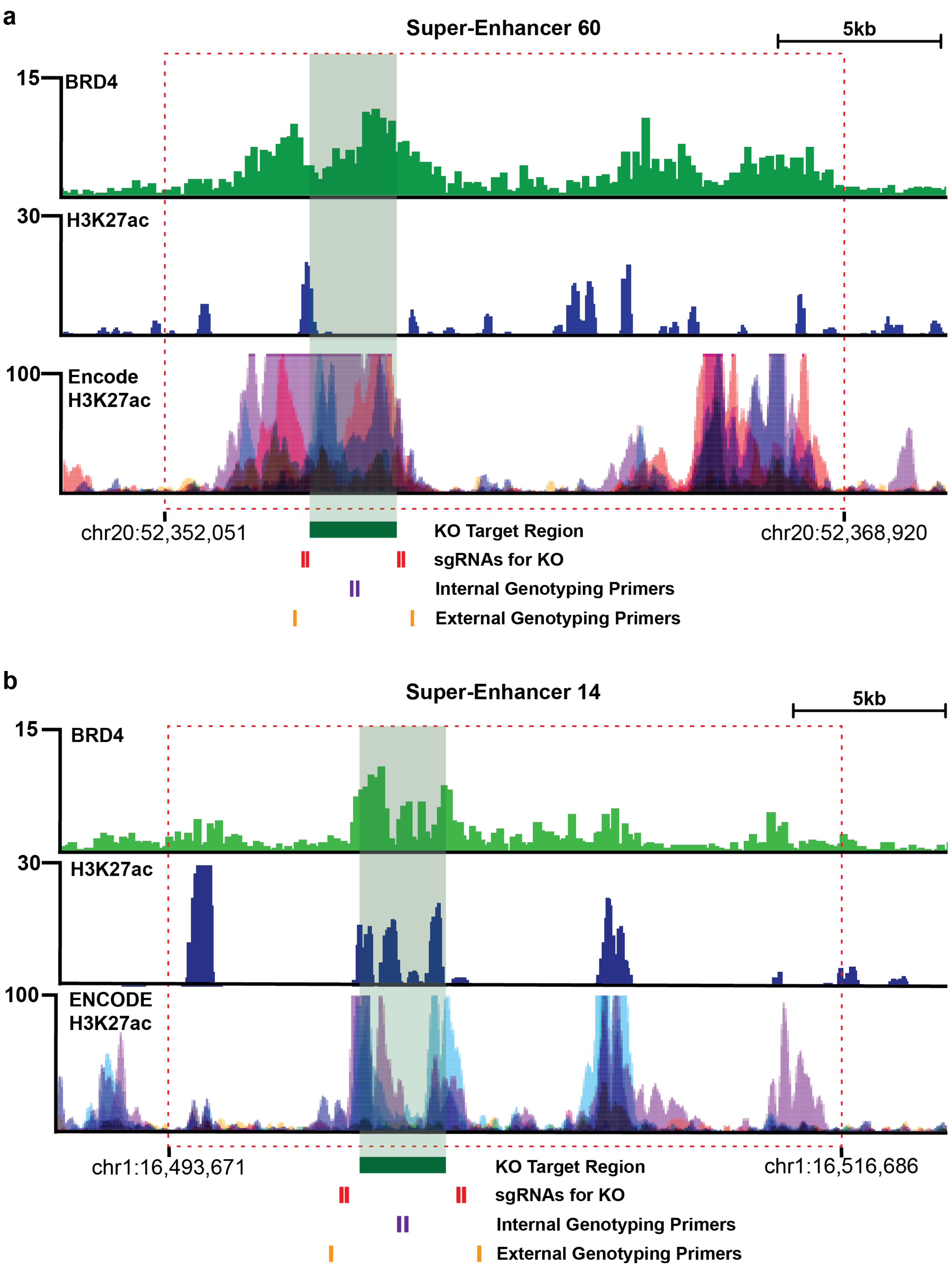

a
Super-Enhancer 60
5kb
15
BRD4
30
H3K27ac
100
Encode
H3K27ac
chr20:52,352,051
KO Target Region
chr20:52,368,920
sgRNAs for KO
Internal Genotyping Primers
External Genotyping Primers
b
Super-Enhancer 14
5kb
15
BRD4
30
H3K27ac
100
ENCODE
H3K27ac
chr1:16,493,671
KO Target Region
chr1:16,516,686
sgRNAs for KO
Internal Genotyping Primers
External Genotyping Primers

Supplementary Figure 5: Super-Enhancer Regions Targetted by CRISPR Knockout

- **a.** Browser track view of SE60 CRISPR KO. Green bar and green shadow indicate the SE target region for KO. Red bars indicate positions of sgRNAs used for CRISPR KO. Purple bars indicate positions of internal genotyping primers. Orange bars indicate positions of external genotyping primers.
- **b.** Browser track view of SE14 CRISPR KO. Green bar and green shadow indicate the SE target region for KO. Red bars indicate positions of sgRNAs used for CRISPR KO. Purple bars indicate positions of internal genotyping primers. Orange bars indicate positions of external genotyping primers.

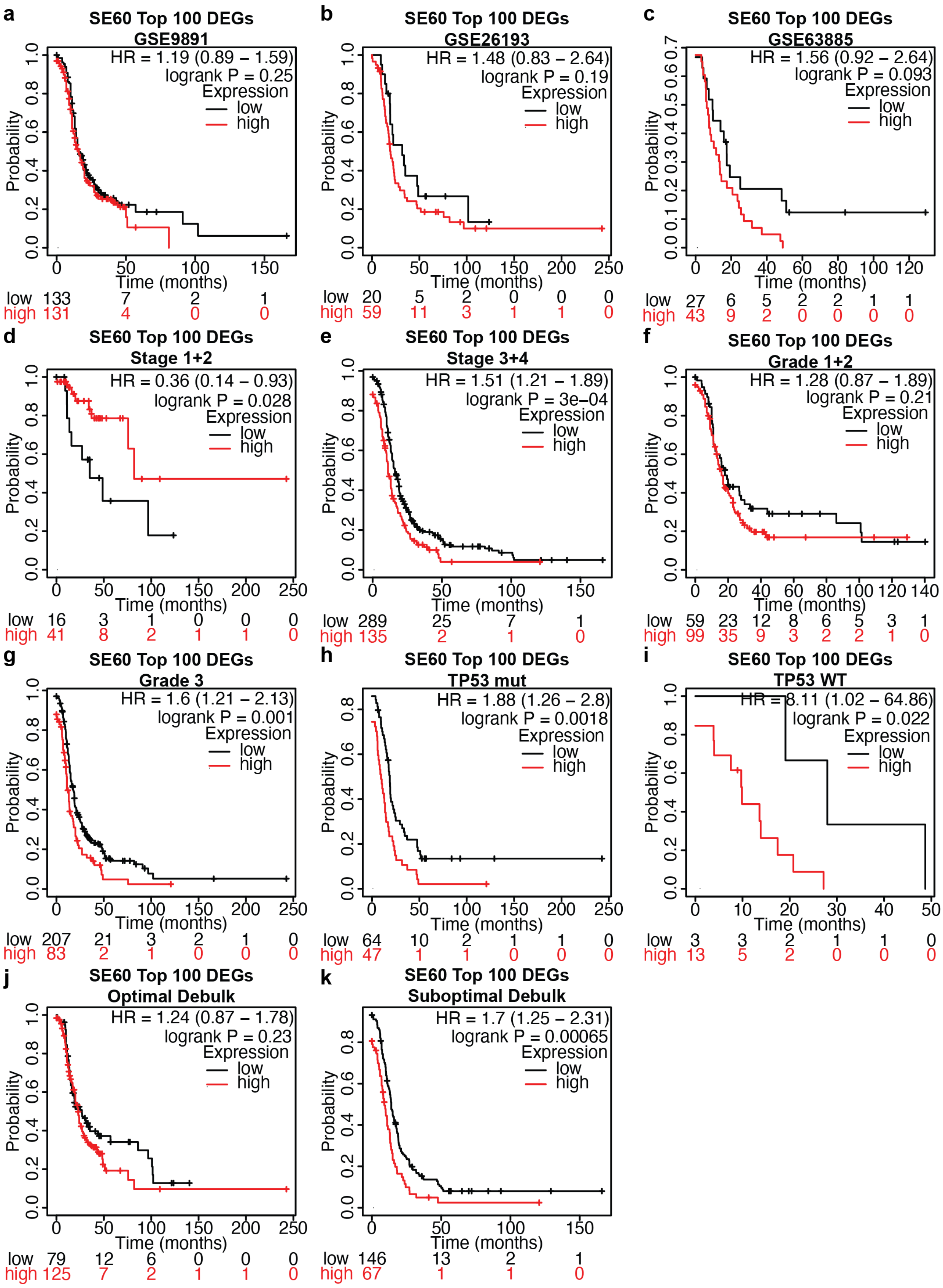

(Supplementary Figure 6 continued on next page)

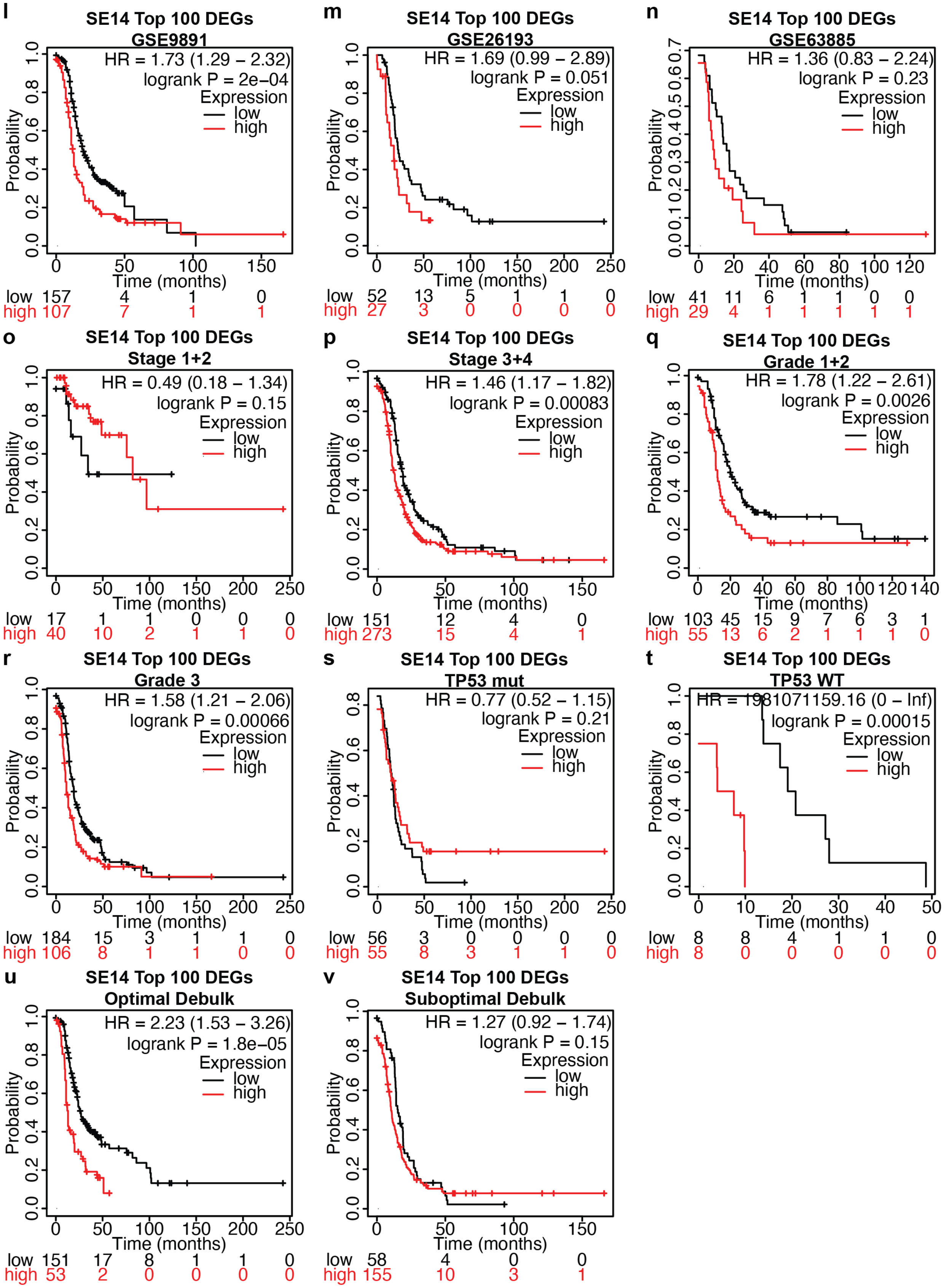

(Supplementary Figure 6 continued on next page)

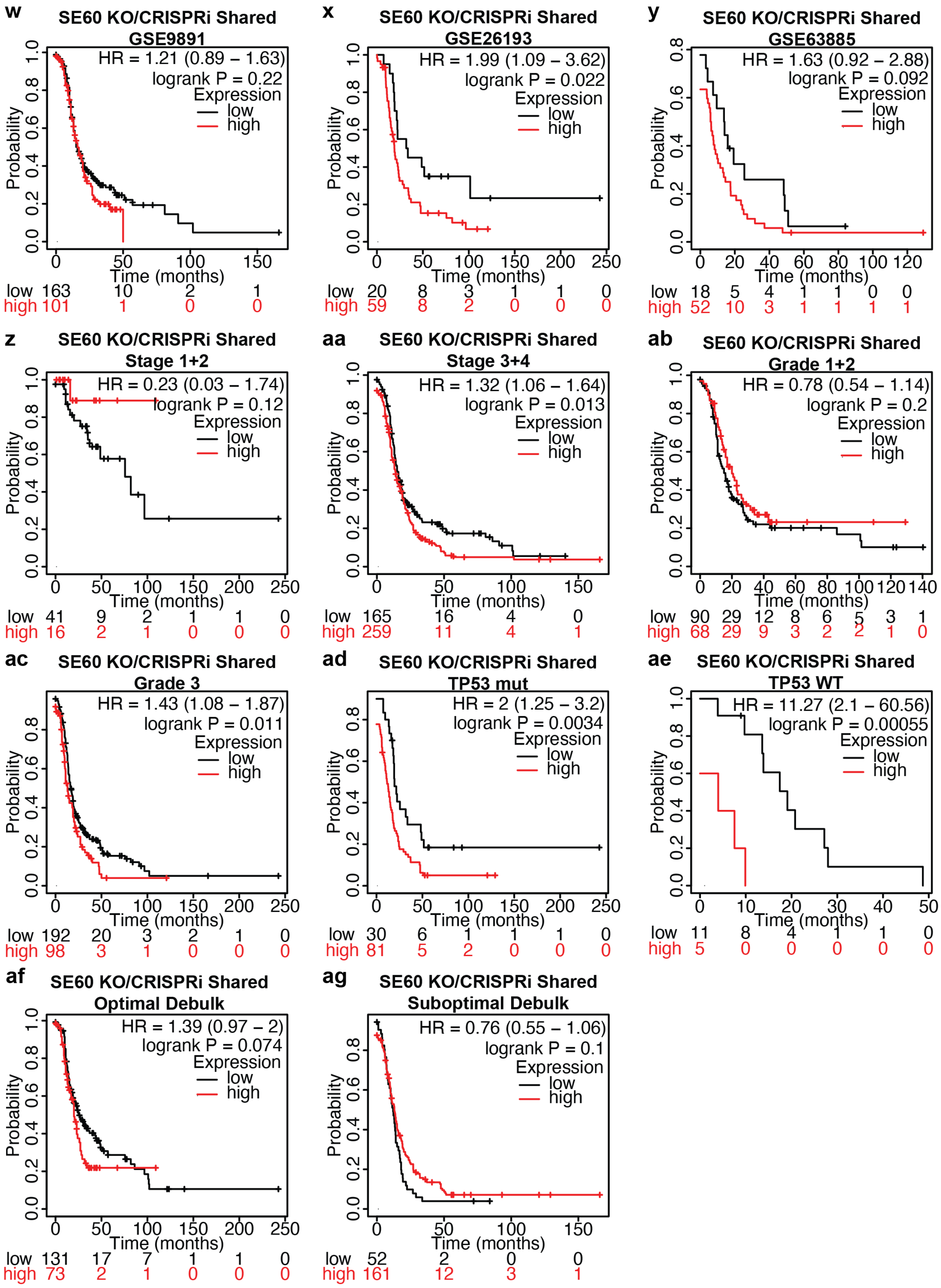

(Supplementary Figure 6 continued on next page)

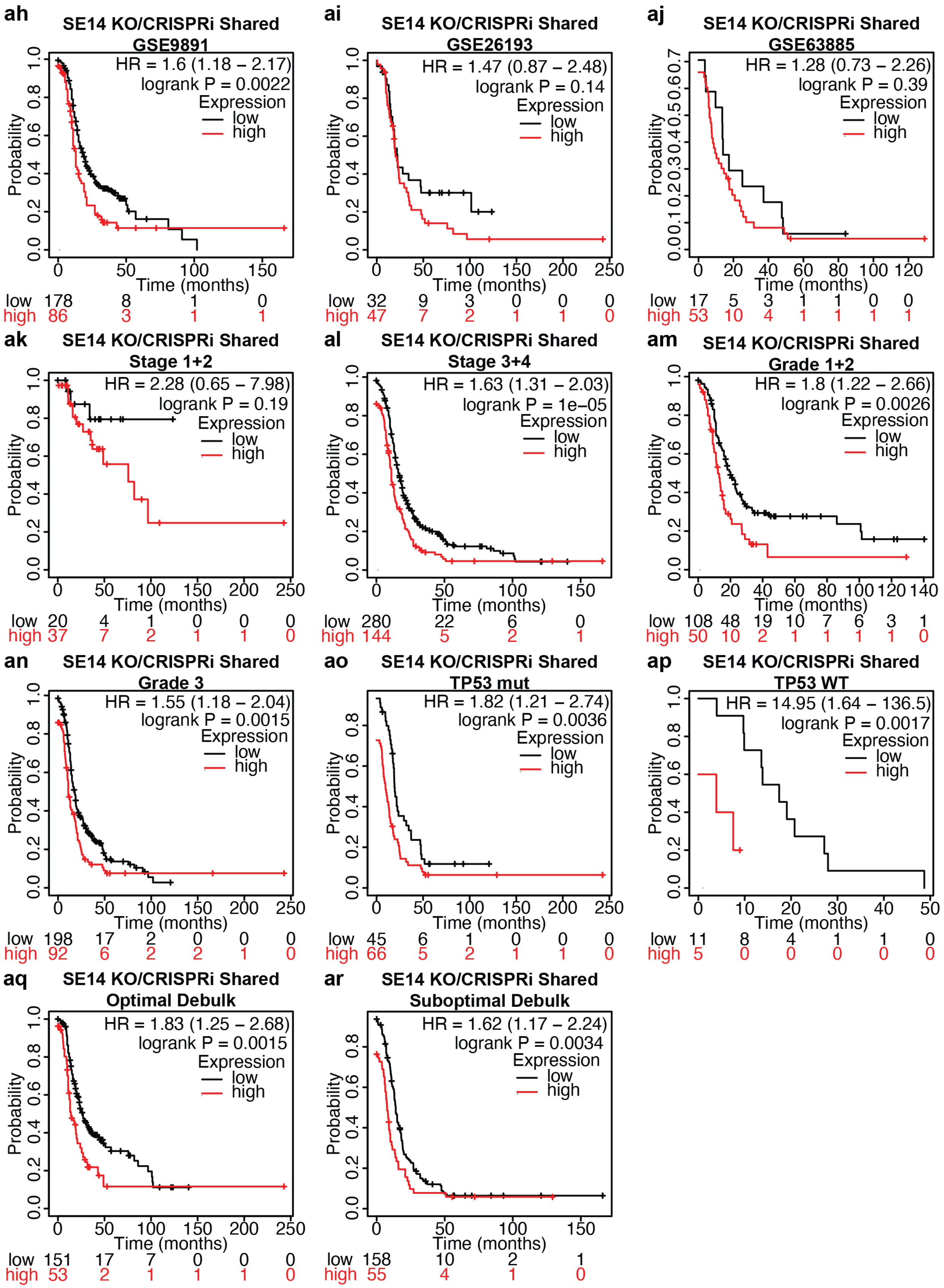

(Supplementary Figure 6 continued on next page)

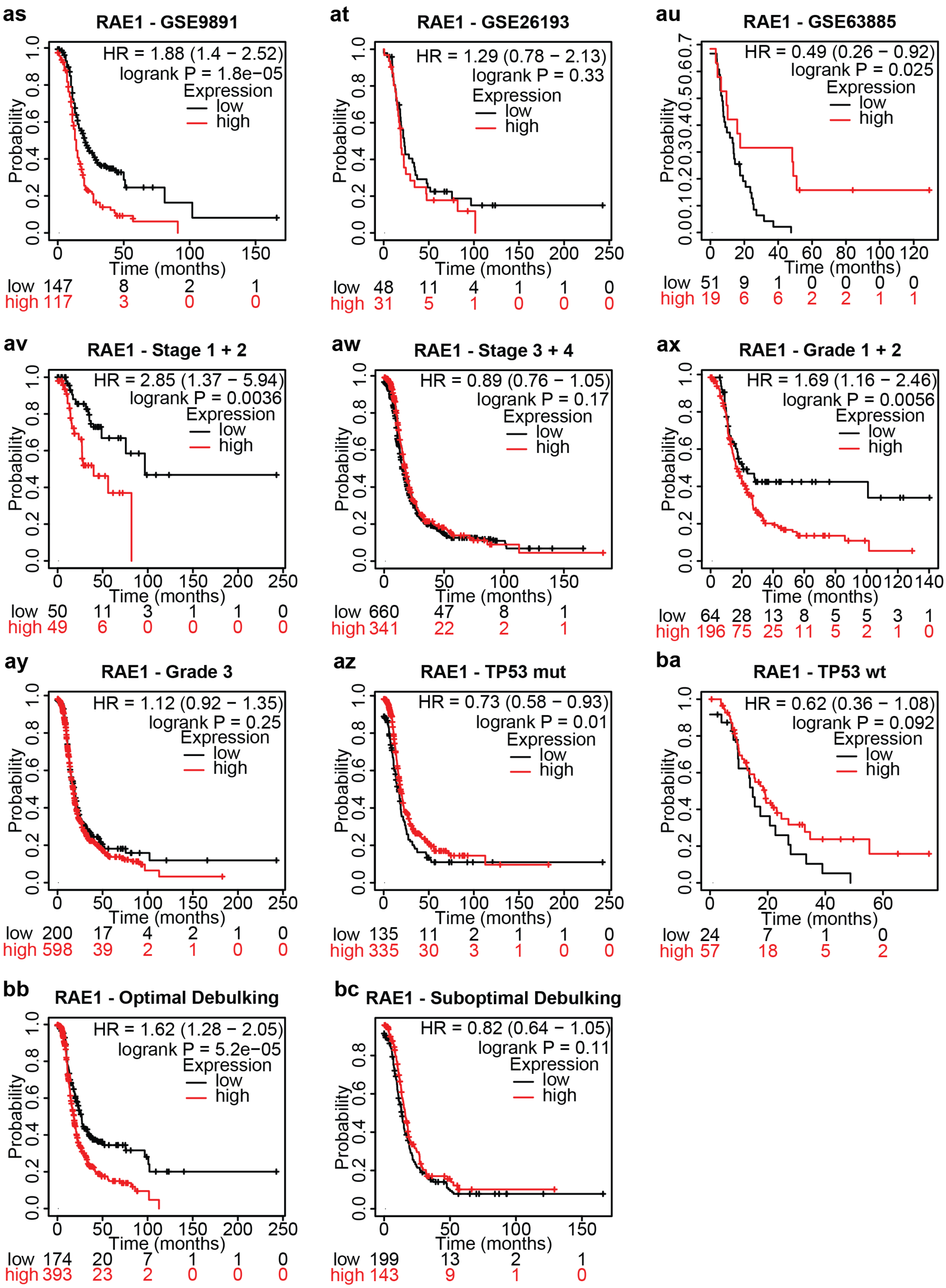

(Supplementary Figure 6 continued on next page)

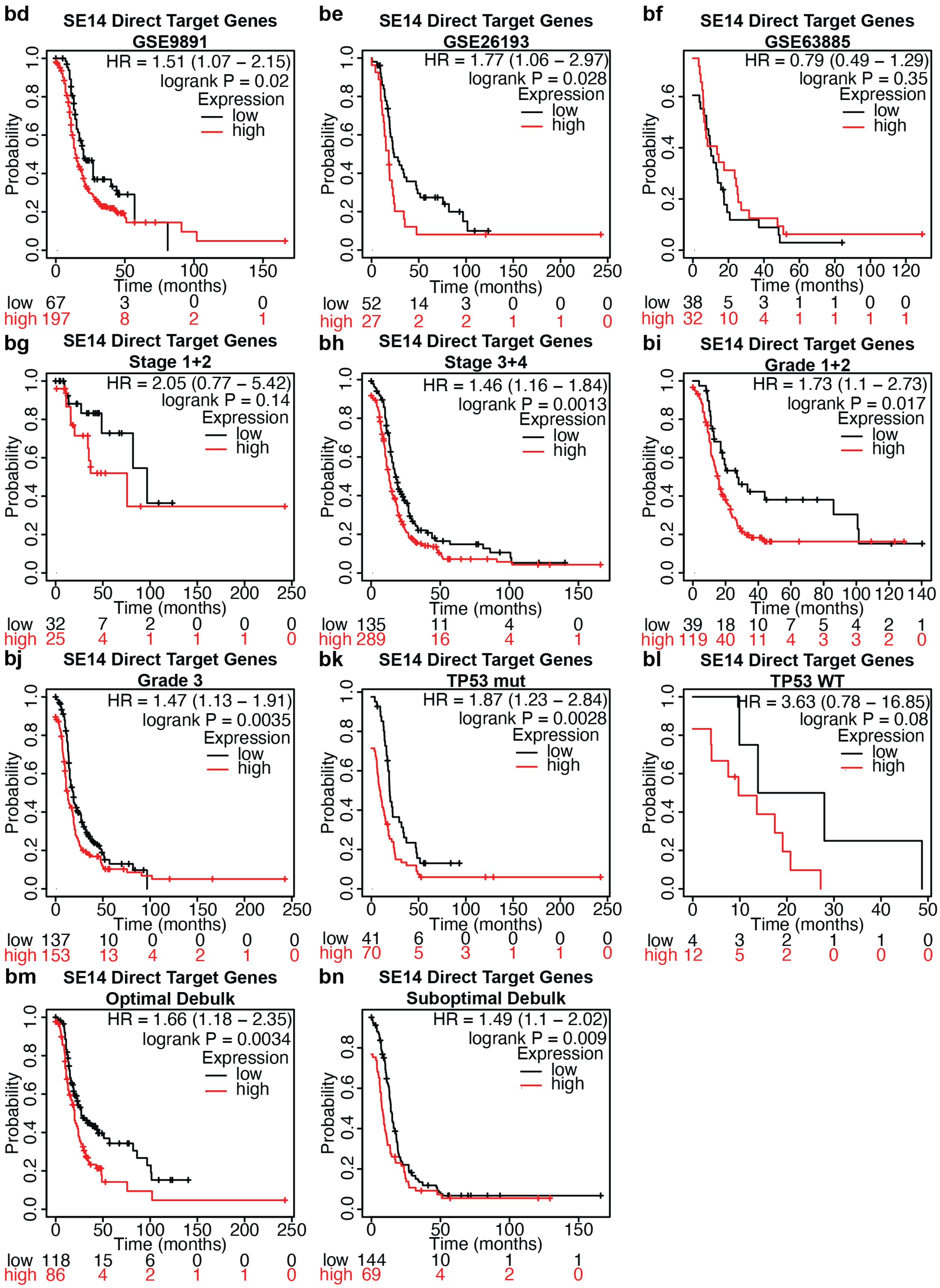

bd
SE14 Direct Target Genes
GSE9891
HR = 1.51 (1.07 − 2.15)
logrank P = 0.02
Expression
low
high
Probability
Time (months)
low 67 3 0 0
high 197 8 2 1
be
SE14 Direct Target Genes
GSE26193
HR = 1.77 (1.06 − 2.97)
logrank P = 0.028
Expression
low
high
Probability
Time (months)
low 52 14 3 0 0 0
high 27 2 2 1 1 0
bf
SE14 Direct Target Genes
GSE63885
HR = 0.79 (0.49 − 1.29)
logrank P = 0.35
Expression
low
high
Probability
Time (months)
low 38 5 3 1 1 0 0
high 32 10 4 1 1 1 1
bg
SE14 Direct Target Genes
Stage 1+2
HR = 2.05 (0.77 − 5.42)
logrank P = 0.14
Expression
low
high
Probability
Time (months)
low 32 7 2 0 0 0
high 25 4 1 1 1 0
bh
SE14 Direct Target Genes
Stage 3+4
HR = 1.46 (1.16 − 1.84)
logrank P = 0.0013
Expression
low
high
Probability
Time (months)
low 135 11 4 0
high 289 16 4 1
bi
SE14 Direct Target Genes
Grade 1+2
HR = 1.73 (1.1 − 2.73)
logrank P = 0.017
Expression
low
high
Probability
Time (months)
low 39 18 10 7 5 4 2 1
high 119 40 11 4 3 3 2 0
bj
SE14 Direct Target Genes
Grade 3
HR = 1.47 (1.13 − 1.91)
logrank P = 0.0035
Expression
low
high
Probability
Time (months)
low 137 10 0 0 0 0
high 153 13 4 2 1 0
bk
SE14 Direct Target Genes
TP53 mut
HR = 1.87 (1.23 − 2.84)
logrank P = 0.0028
Expression
low
high
Probability
Time (months)
low 41 6 0 0 0 0
high 70 5 3 1 1 0
bl
SE14 Direct Target Genes
TP53 WT
HR = 3.63 (0.78 − 16.85)
logrank P = 0.08
Expression
low
high
Probability
Time (months)
low 4 3 2 1 1 0
high 12 5 2 0 0 0
bm
SE14 Direct Target Genes
Optimal Debulk
HR = 1.66 (1.18 − 2.35)
logrank P = 0.0034
Expression
low
high
Probability
Time (months)
low 118 15 6 0 0 0
high 86 4 2 1 1 0
bn
SE14 Direct Target Genes
Suboptimal Debulk
HR = 1.49 (1.1 − 2.02)
logrank P = 0.009
Expression
low
high
Probability
Time (months)
low 144 10 1 1
high 69 4 2 0

Supplementary Figure 6: Further Kaplan Meier Survival Analysis of Geneset Subsetting By Dataset and Clinical Variable.

a-k: Kaplan Meier Survival Analysis of the top 100 DEGs for SE60 using patient/clinical data from individual datasets GSE891[4], GSE63885[5], GSE26193[6] (datasets available through KM Plot tool[7,8]), all patients who were Stage 1+2, all patients who were Stage 3+4, all patients who were Grade 1+2, all patients who were Grade 3, all patients with mutant TP53, all patients with WT TP53, all patients with optimal debulking, and all patients with suboptimal debulking.

i-v: Kaplan Meier Survival Analysis of the top 100 DEGs for SE14 using patient/clinical data from individual datasets GSE891[4], GSE63885[5], GSE26193[6], all patients who were Stage 1+2, all patients who were Stage 3+4, all patients who were Grade 1+2, all patients who were Grade 3, all patients with mutant TP53, all patients with WT TP53, all patients with optimal debulking, and all patients with suboptimal debulking.

w-ag: Kaplan Meier Survival Analysis of the downregulated DEGs for SE60 idenfitied using both CRISPR-KO and CRISPRi using patient/clinical data from individual datasets GSE891[4], GSE63885[5], GSE26193[6], all patients who were Stage 1+2, all patients who were Stage 3+4, all patients who were Grade 1+2, all patients who were Grade 3, all patients with mutant TP53, all patients with WT TP53, all patients with optimal debulking, and all patients with suboptimal debulking.

ah-ar: Kaplan Meier Survival Analysis of the downregulated DEGs for SE14 idenfitied using both CRISPR-KO and CRISPRi using patient/clinical data from individual datasets GSE891[4], GSE63885[5], GSE26193[6], all patients who were Stage 1+2, all patients who were Stage 3+4, all patients who were Grade 1+2, all patients who were Grade 3, all patients with mutant TP53, all patients with WT TP53, all patients with optimal debulking, and all patients with suboptimal debulking.

as-bc: Kaplan Meier Survival Analysis of the direct targets of SE60 using patient/clinical data from individual datasets GSE891[4], GSE63885[5], GSE26193[6], all patients who were Stage 1+2, all patients who were Stage 3+4, all patients who were Grade 1+2, all patients who were Grade 3, all patients with mutant TP53, all patients with WT TP53, all patients with optimal debulking, and all patients with suboptimal debulking.

bd-bn: Kaplan Meier Survival Analysis of the direct targets of SE14 using patient/clinical data from individual datasets GSE891[4], GSE63885[5], GSE26193[6], all patients who were Stage 1+2, all patients who were Stage 3+4, all patients who were Grade 1+2, all patients who were Grade 3, all patients with mutant TP53, all patients with WT TP53, all patients with optimal debulking, and all patients with suboptimal debulking.

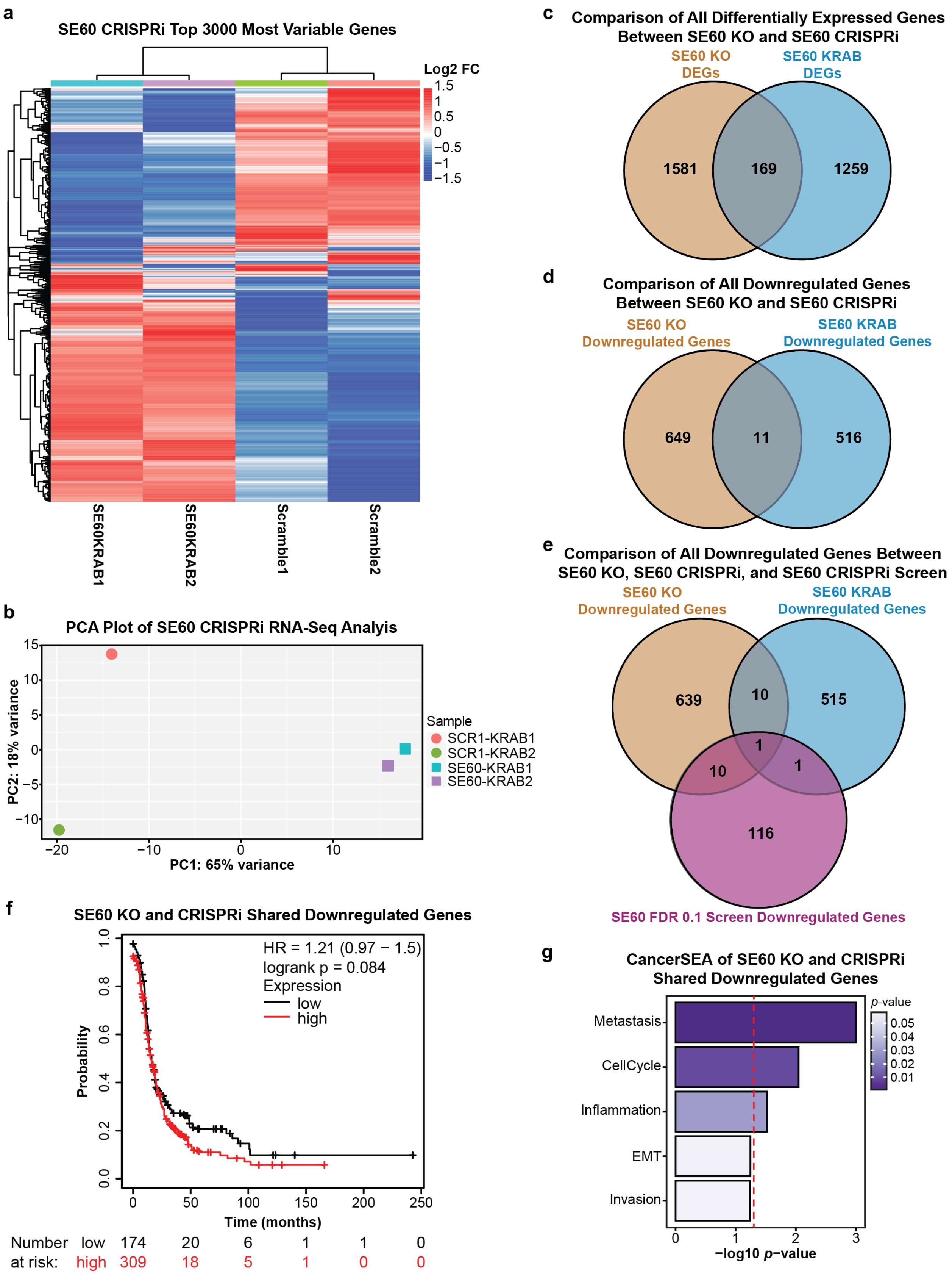
a
SE60 CRISPRi Top 3000 Most Variable Genes
Log2 FC
1.5
1
0.5
0
−0.5
−1
−1.5
SE60KRAB1
SE60KRAB2
Scramble1
Scramble2
b
PCA Plot of SE60 CRISPRi RNA-Seq Analyis
PC2: 18% variance
PC1: 65% variance
Sample
SCR1-KRAB1
SCR1-KRAB2
SE60-KRAB1
SE60-KRAB2
c
Comparison of All Differentially Expressed Genes
Between SE60 KO and SE60 CRISPRi
SE60 KO DEGs
SE60 KRAB DEGs
1581
169
1259
d
Comparison of All Downregulated Genes
Between SE60 KO and SE60 CRISPRi
SE60 KO Downregulated Genes
SE60 KRAB Downregulated Genes
649
11
516
e
Comparison of All Downregulated Genes Between
SE60 KO, SE60 CRISPRi, and SE60 CRISPRi Screen
SE60 KO Downregulated Genes
SE60 KRAB Downregulated Genes
639
10
515
1
10
1
116
SE60 FDR 0.1 Screen Downregulated Genes
f
SE60 KO and CRISPRi Shared Downregulated Genes
HR = 1.21 (0.97 − 1.5)
logrank p = 0.084
Expression
low
high
Probability
Time (months)
Number at risk: low 174 20 6 1 1 0
high 309 18 5 1 0 0
g
CancerSEA of SE60 KO and CRISPRi
Shared Downregulated Genes
Metastasis
CellCycle
Inflammation
EMT
Invasion
p-value
0.05
0.04
0.03
0.02
0.01
−log10 p−value

Supplementary Figure 7: Comparison Between CRISPRi RNA-Seq and CRISPR-KO RNA-Seq for SE60 Further Suggests Involvement in Metastasis

- **a.** Unsupervised hierarchical clustering heatmap of the top 3000 most variable genes between scramble controls and SE60 CRISPRi cells measured by RNA-seq.
- **b.** PCA plot showing the variance landscape of both scramble controls and the two SE60 CRISPRi RNA-seq replicates.
- **c.** Overlap of all DEGs detected by CRISPR-KO (left) and CRISPRi (right) targeting SE60. CRISPRi DEGS detected at cutoffs +/- 0.5 Fold Change and adjusted *p*-value of 0.05.
- **d.** Overlap of all downregulated DEGs (potential targets) detected by CRISPR-KO (left) and CRISPRi (right) targeting SE60.
- **e.** Overlap of all downregulated DEGs (potential target genes) detected by CRISPR-KO (left) and CRISPRi (right) targeting SE60 as well as the CRISPRi Screen (bottom).
- **f.** Kaplan Meier plot generated using the KM Plot tool[7,8] showing the clinical significance (HR = hazard ratio) of the SE60 KO/CRISPRi shared downregulated DEGS (as noted in D), the red line denotes patients with high expression of this gene signature, significant *p*-values are denoted in red text.
- **g.** CancerSEA gene pathway analysis of the SE60 KO/CRISPRi shared downregulated DEGS (as determined by Kolmogorov-Smirnov style GSVA followed by Spearman's rank test with BH FDR correction). The red line denotes the metric for a *p*-value of 0.05 (significance) converted into the -log10 scale, anything past the line is a significant term.

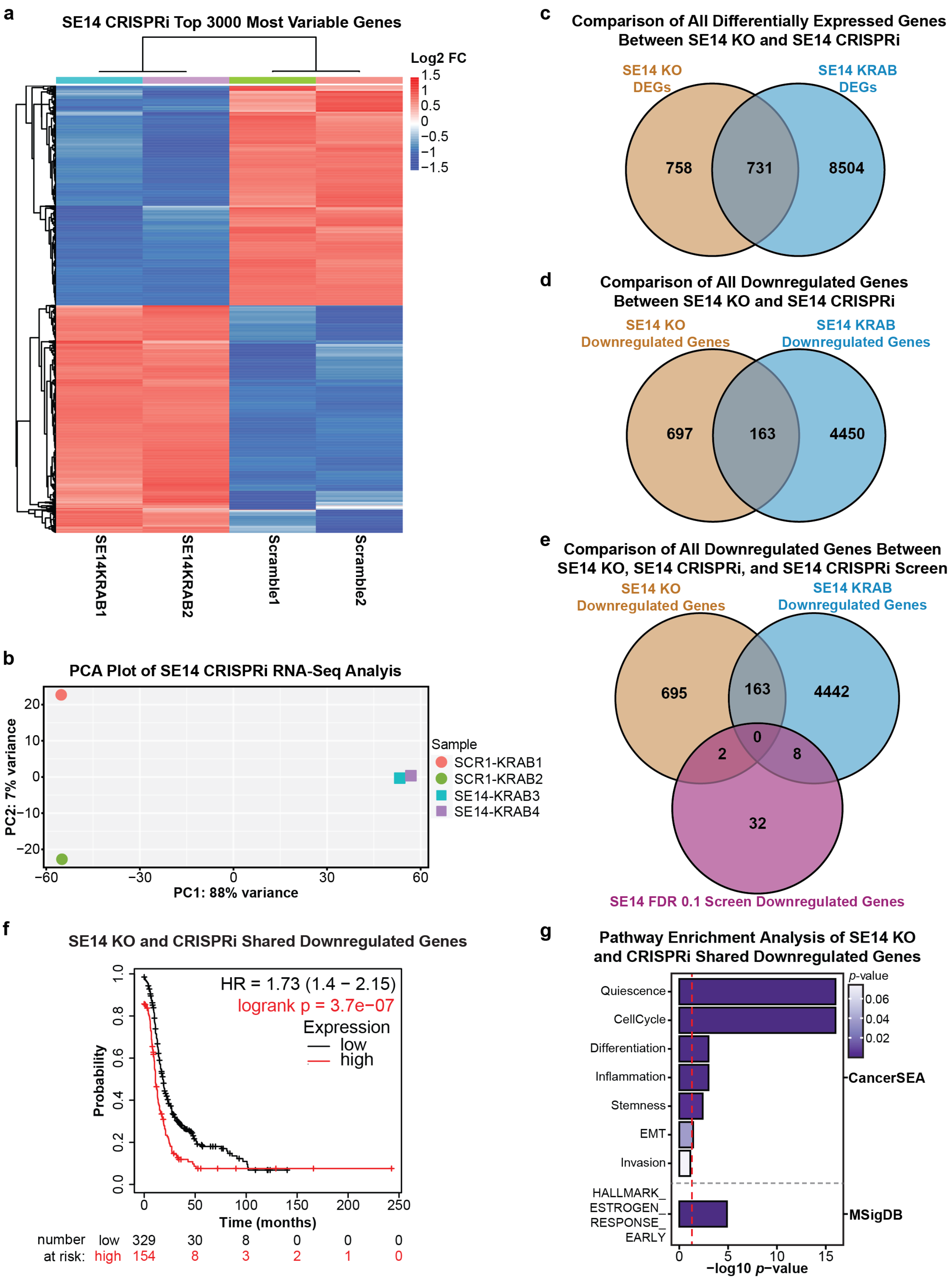

a
SE14 CRISPRi Top 3000 Most Variable Genes
Log2 FC
1.5
1
0.5
0
−0.5
−1
−1.5
SE14KRAB1
SE14KRAB2
Scramble1
Scramble2
b
PCA Plot of SE14 CRISPRi RNA-Seq Analyis
PC2: 7% variance
PC1: 88% variance
Sample
SCR1-KRAB1
SCR1-KRAB2
SE14-KRAB3
SE14-KRAB4
c
Comparison of All Differentially Expressed Genes Between SE14 KO and SE14 CRISPRi
SE14 KO DEGs
SE14 KRAB DEGs
758
731
8504
d
Comparison of All Downregulated Genes Between SE14 KO and SE14 CRISPRi
SE14 KO Downregulated Genes
SE14 KRAB Downregulated Genes
697
163
4450
e
Comparison of All Downregulated Genes Between SE14 KO, SE14 CRISPRi, and SE14 CRISPRi Screen
SE14 KO Downregulated Genes
SE14 KRAB Downregulated Genes
695
163
4442
0
2
8
32
SE14 FDR 0.1 Screen Downregulated Genes
f
SE14 KO and CRISPRi Shared Downregulated Genes
HR = 1.73 (1.4 − 2.15)
logrank p = 3.7e−07
Expression
low
high
Probability
Time (months)
number at risk: low 329 30 8 0 0 0
high 154 8 3 2 1 0
g
Pathway Enrichment Analysis of SE14 KO and CRISPRi Shared Downregulated Genes
Quiescence
CellCycle
Differentiation
Inflammation
Stemness
EMT
Invasion
HALLMARK_ESTROGEN_RESPONSE_EARLY
p-value
0.06
0.04
0.02
CancerSEA
MSigDB
−log10 p−value

Supplementary Figure 8: Comparison Between CRISPRi RNA-seq and CRISPR-KO RNA-seq for SE14 Further Suggests Involvement in Metastasis

- **a.** Unsupervised hierarchical clustering heatmap of the top 3000 most variable genes between scramble controls and SE14 CRISPRi cells measured by RNA-seq.
- **b.** PCA plot showing the variance landscape of both scramble controls and the two SE14 CRISPRi RNA-seq replicates
- **c.** Overlap of all DEGs detected by CRISPR-KO (left) and CRISPRi (right) targeting SE14; CRISPRi DEGS detected at cutoffs +/- 0.5 Fold Change and adjusted *p*-value of 0.05.
- **d.** Overlap of all downregulated DEGs (potential targets) detected by CRISPR-KO (left) and CRISPRi (right) targeting SE14.
- **e.** Overlap of all downregulated DEGs (potential target genes) detected by CRISPR-KO (left) and CRISPRi (right) targeting SE14 as well as the CRISPRi Screen (bottom).
- **f.** Kaplan Meier plot generated using the KM Plot tool[7,8] showing the clinical significance (HR = hazard ratio) of the SE14 KO/CRISPRi shared downregulated DEGS (as noted in D), the red line denotes patients with high expression of this gene signature, significant *p*-values are denoted in red text.
- **g.** CancerSEA gene pathway analysis of the SE14 KO/CRISPRi shared downregulated DEGS (as determined by Kolmogorov-Smirnov style GSVA followed by Spearman's rank test with BH FDR correction). The red line denotes the metric for a *p*-value of 0.05 (significance) converted into the -log10 scale, anything past the line is a significant term.

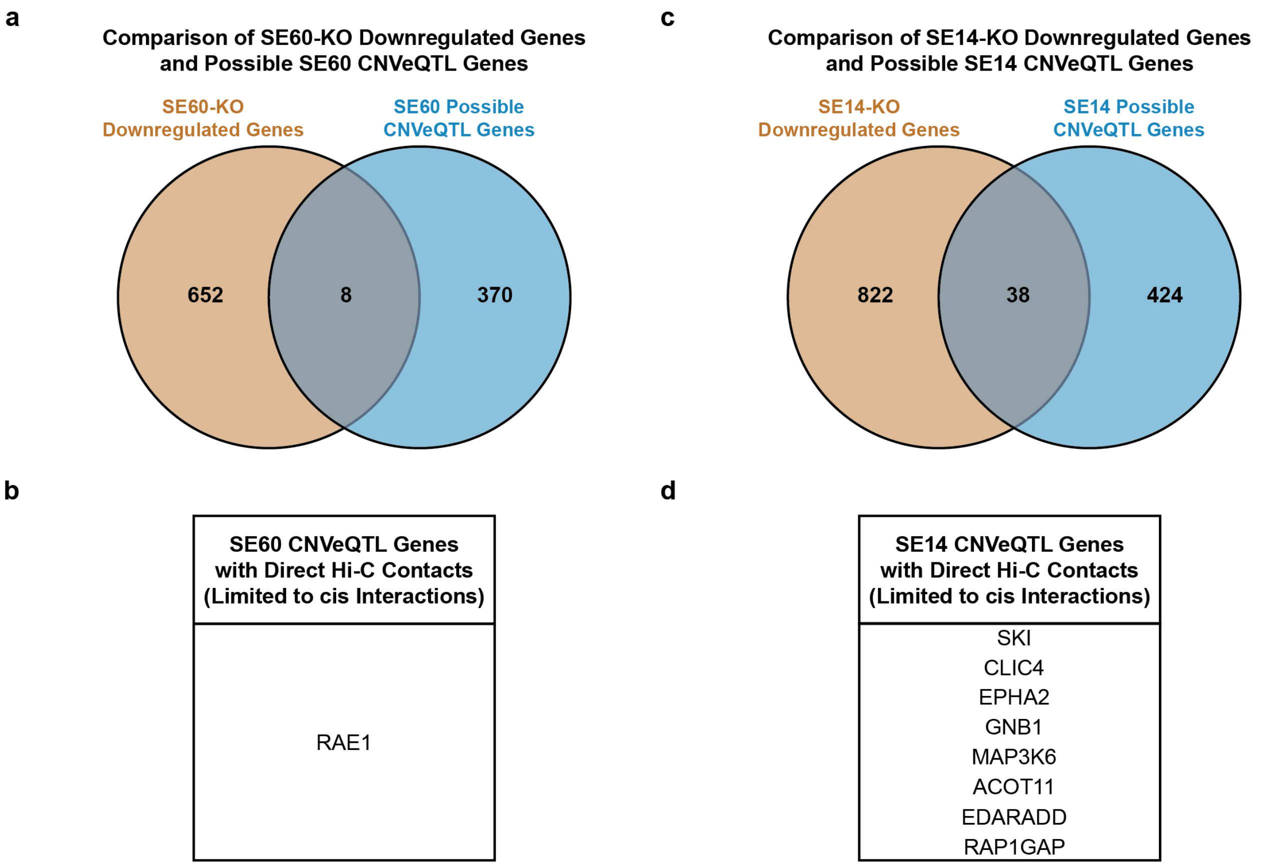

Supplementary Figure 9: Hi-C Validated *cis* Direct Target Genes are Validated by CNVeQTL Predictions

- **a.** Venn diagram depicting the results of the overlap analysis between Hi-C validated *cis* direct target genes detected from SE60 KO and all possible *cis* CNVeQTLs.
- **b.** Table showing the results of overlapping the shared *cis* genes with HI-C *cis*-direct targets, one gene RAE1 was predicted via CNVeQTL analysis
- **c.** Venn Diagram depicting the results of the overlap between *cis*-genes detected from the SE14 KO and all possible *cis*-CNVeQTLs.
- **d.** Table showing the results of overlapping the shared *cis* genes with HI-C *cis*-direct targets, eight genes were predicted via CNVeQTL analysis

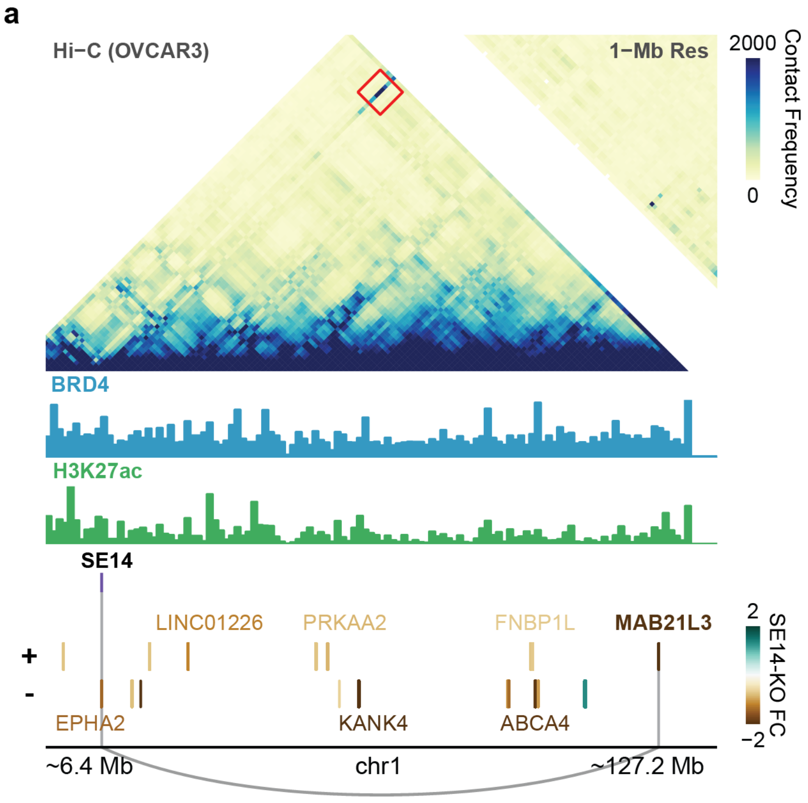

Supplementary Figure 10: Hi-C Analysis Identifies Strong Long Range Direct Interaction Between SE14 and *MAB21L3*

a. Hi-C contact heatmap showing the interaction between *MAB21L3*, a direct target of SE14, and the SE locus itself (*red square*) across a vast distance.

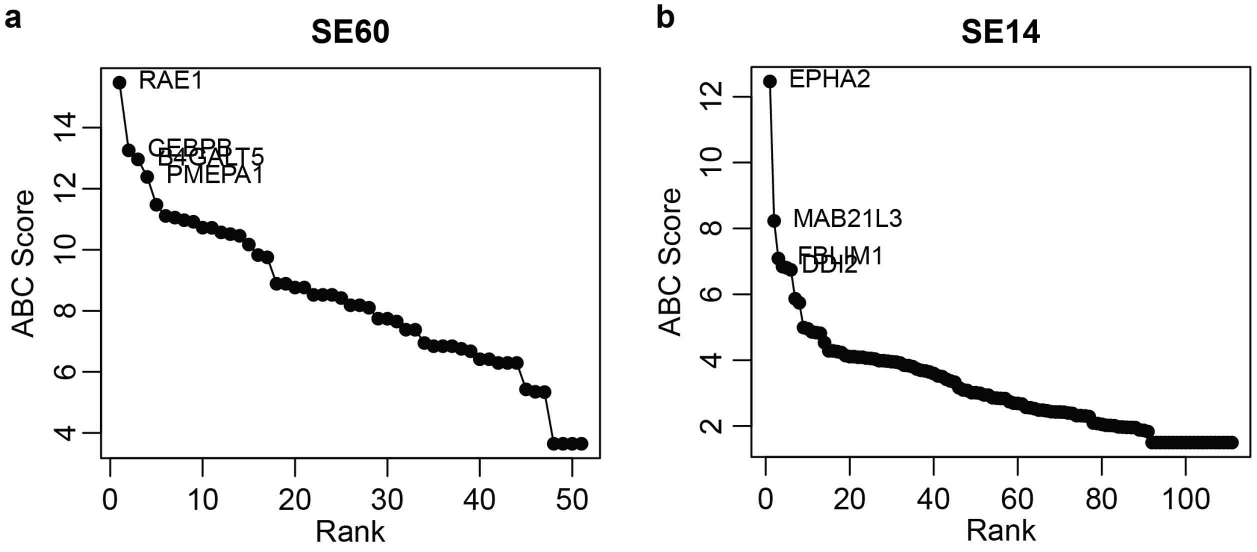

Supplementary Figure 11: Activity-by-Contact (ABC) Scores for all Expressed *Cis*-Genes in OVCAR3 for SE14 and SE60

- **a.** ABC scores for all *cis*-genes nominally expressed in OVCAR3 and the SE60 locus, the top four genes by ABC score are given.
- **b.** ABC scores for all *cis*-genes nominally expressed in OVCAR3 and the SE14 locus, the top four genes by ABC score are given.

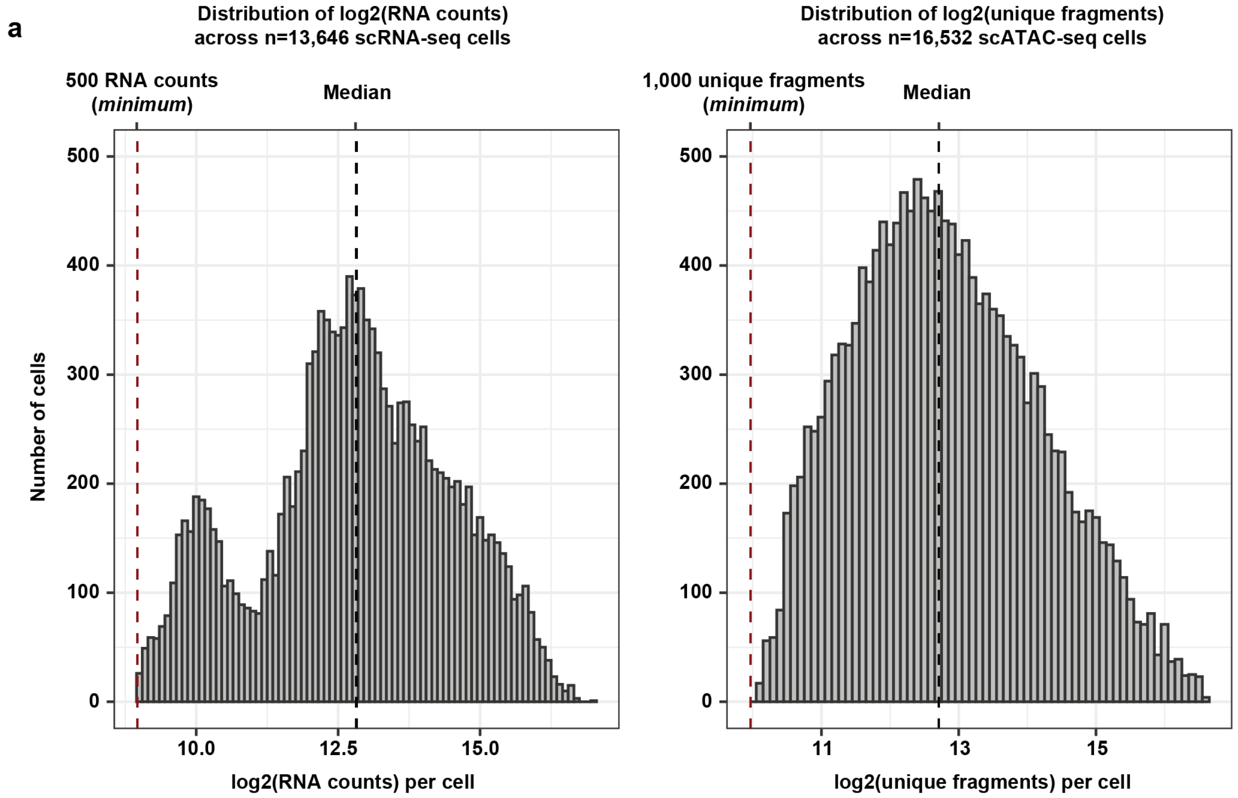

Supplementary Figure 12: High Quality scRNA-seq and scATAC-seq Data in Terms of RNA Counts Per Cell and Unique Fragments Per Cell

a. Histogram of log2(RNA counts) across 13,646 scRNA-seq cells from both patient samples (*left*). Histogram of log2(unique fragments) across 16,532 scATAC-seq cells from both patient samples (*right*). The dashed red lines represent the standard minimums of 500 RNA counts per cell and 1,000 unique fragments per cell in scRNA-seq and scATAC-seq, respectively. The dashed black lines represent the median values of RNA counts per cell (median=7,234) and unique fragments per cell (median=6,707) in scRNA-seq and scATAC-seq, respectively.

KO guide target	Forward Sequence	Reverse Sequence	Genomic Coordinates
SE14_LFT_INNR	CACCGAGCGCCTTTCTGAGCGTCAC	AAACGTGACGCTCAGAAAGGCGCTC	chr1:16,499,731-16,499,750
SE14_LFT_OUTR	CACCGACCCTTCAAGTGACCTCGGC	AAACGCCGAGGTCACTTGAAGGGTC	chr1:16,499,554-16,499,573
SE14_RGT_OUTR	CACCGTGCTAAAAGGCTCCCGTAGT	AAACACTACGGGAGCCTTTTAGCAC	chr1:16,502,388-16,502,407
SE14_RGT_INNR	CACCGATTTGAGACCTAATCAGCGG	AAACCCGCTGATTAGGTCTCAAATC	chr1:16,502,255-16,502,274
SE60_LFT_OUT	CACCGTTACACAGAAGGCCGTTCAC	AAACGTGAACGGCCTTCTGTGTAAC	chr20:52,355,052-52,355,071
SE60_LFT_INNR	CACCGAAGTGCAGCTCAACTGCGCA	AAACTGCGCAGTTGAGCTGCACTTC	chr20:52,355,148-52,355,167
SE60_RGT_INNR	CACCGATCGAACTCCTCCCTCGGGA	AAACTCCCGAGGGAGGAGTTCGATC	chr20:52,356,832-52,356,851
SE60_RGT_OUTR	CACCGGTGAAATCATGTATACGAC	AAACGTCGTATACATGATTTCACC	chr20:52,356,874-52,356,893

Supplementary Table 1. Oligonucleotide sgRNA sequences for CRISPR Cas9 Super-Enhancer Deletion. Sequences are listed 5’ to 3’.

Primer Name	Sequence	Genomic Coordinates
SE14-Internal-F	GGTGAAGGGAGAATAGCGGTAG	chr1:16,500,471-16,500,492
SE14-Internal-R	CTGACTGCCTGATGGGGTTG	chr1:16,501,022-16,501,041
SE14-External-F	GTGGCTCACCCTTGTAATCTCA	chr1:16,499,180-16,499,201
SE14-External-R	AGAGAGAGAGAGACAGAGGCAG	chr1:16,502,806-16,502,827
SE60-Internal-F	GCAGCTCATGATCTCACCAGAG	chr20:52,355,674-52,355,695
SE60-Internal-R	AGAAGCAAACACTGAAAGCCAC	chr20:52,356,236-52,356,257
SE60-External-F	TCCCGCATCATTTTCACACATG	chr20:52,354,875-52,354,896
SE60-External-R	ATTTCCTTCTTCCCAGGCACAA	chr20:52,356,962-52,356,983

Supplementary Table 2. Super-Enhancer Deletion Screening Primers. Sequences are listed 5' to 3'.

References

1. Regner, M.J. *et al.* A multi-omic single-cell landscape of human gynecologic malignancies. *Mol Cell* (2021).
2. Coetzee, S.G. *et al.* Cell-type-specific enrichment of risk-associated regulatory elements at ovarian cancer susceptibility loci. *Human molecular genetics* **24**, 3595-3607 (2015).
3. Hnisz, D. *et al.* Super-enhancers in the control of cell identity and disease. *Cell* **155**, 934-47 (2013).
4. Tothill, R.W. *et al.* Novel molecular subtypes of serous and endometrioid ovarian cancer linked to clinical outcome. *Clin Cancer Res* **14**, 5198-208 (2008).
5. Lisowska, K.M. *et al.* Gene expression analysis in ovarian cancer - faults and hints from DNA microarray study. *Front Oncol* **4**, 6 (2014).
6. Mateescu, B. *et al.* miR-141 and miR-200a act on ovarian tumorigenesis by controlling oxidative stress response. *Nat Med* **17**, 1627-35 (2011).
7. Gyorffy, B., Lánczky, A. & Szállási, Z. Implementing an online tool for genome-wide validation of survival-associated biomarkers in ovarian-cancer using microarray data from 1287 patients. *Endocr Relat Cancer* **19**, 197-208 (2012).
8. Lánczky, A. & Győrffy, B. Web-Based Survival Analysis Tool Tailored for Medical Research (KMplot): Development and Implementation. *J Med Internet Res* **23**, e27633 (2021).
