# Supplementary material for: A multi-omic dissection of super-enhancer driven oncogenic gene expression programs in ovarian cancer
Source: Nat Commun. 2022 Jul 22;13:4247. doi: 10.1038/s41467-022-31919-8 (PMC9307778; doi:10.1038/s41467-022-31919-8)
Supplement: Supplementary file 2 — Description of Additional Supplementary Files [file 41467_2022_31919_MOESM2_ESM.pdf]

Description of Additional Supplementary Data File

Supplementary Data 1 Description: This file contains all of the SE hg19 coordinates, CRISPRi sgRNA sequences, and CRISPRi Screen cluster identity.

Supplementary Data 2 Description: This file contains all of the hg19 H3K27ac peak coordinates.

Supplementary Data 3 Description: This file contains all of the hg19 BRD4 peak coordinates.

Supplementary Data 4 Description: This file contains all of the coaccessible H3K27ac/BRD4 peaks used to identify SEs via the ROSE pipeline.

Supplementary Data 5 Description: This file contains all of the CNVeQTL identified using Matrix eQTL with a p-value cutoff of 1e-3.

Supplementary Data 6 Description: This file contains all of the genes identified as differentially expressed via the CRISPRi Screen analysis at an FDR of 0.1.

Supplementary Data 7 Description: This file contains all of the DEGs detected by DESEQ2 in all CRISPRKO/CRISPRi experiments.

Supplementary Data 8 Description: This file contains all of the cisDEGs detected from the CRISPR-KO analysis with information relaying whether or not they are direct or indirect targets as determined by Hi-C.

Supplementary Data 9 Description: This file contains a list of cisgenes for both super-enhancer 14 and superenhancer 60 listed with their ABC contact scores.

Supplementary Data 10 Description: This file contains the summary of all additional clinical analysis of the gene-sets identified in this paper and shown in Supplementary figure 6.
