# Supplementary material for: A multi-omic dissection of super-enhancer driven oncogenic gene expression programs in ovarian cancer
Source: Nat Commun. 2022 Jul 22;13:4247. doi: 10.1038/s41467-022-31919-8 (PMC9307778; doi:10.1038/s41467-022-31919-8)
Supplement: Supplementary file 13 — Reporting Summary [file 41467_2022_31919_MOESM13_ESM.pdf]

nature portfolio

Corresponding author(s): Hector L. Franco

Last updated by author(s): Jul 5, 2022

Reporting Summary

Nature Portfolio wishes to improve the reproducibility of the work that we publish. This form provides structure for consistency and transparency in reporting. For further information on Nature Portfolio policies, see our Editorial Policies and the Editorial Policy Checklist.

Statistics

For all statistical analyses, confirm that the following items are present in the figure legend, table legend, main text, or Methods section.

n/a	Confirmed	
☐	☒	The exact sample size (*n*) for each experimental group/condition, given as a discrete number and unit of measurement
☐	☒	A statement on whether measurements were taken from distinct samples or whether the same sample was measured repeatedly
☐	☒	The statistical test(s) used AND whether they are one- or two-sided<br>*Only common tests should be described solely by name; describe more complex techniques in the Methods section.*
☒	☐	A description of all covariates tested
☐	☒	A description of any assumptions or corrections, such as tests of normality and adjustment for multiple comparisons
☐	☒	A full description of the statistical parameters including central tendency (e.g. means) or other basic estimates (e.g. regression coefficient) AND variation (e.g. standard deviation) or associated estimates of uncertainty (e.g. confidence intervals)
☐	☒	For null hypothesis testing, the test statistic (e.g. *F*, *t*, *r*) with confidence intervals, effect sizes, degrees of freedom and *P* value noted<br>*Give P values as exact values whenever suitable.*
☒	☐	For Bayesian analysis, information on the choice of priors and Markov chain Monte Carlo settings
☒	☐	For hierarchical and complex designs, identification of the appropriate level for tests and full reporting of outcomes
☐	☒	Estimates of effect sizes (e.g. Cohen's *d*, Pearson's *r*), indicating how they were calculated

Our web collection on statistics for biologists contains articles on many of the points above.

Software and code

Policy information about availability of computer code

Data collection

Raw data from publicly available sources (GEO/SRA) was downloaded using sratoolkit fastq-dump, version=2.9.0.
No software was used to download the TCGA data or any data generated by our lab for the purpose of this study.

Data analysis

Custom code used to analyze data in this project are located at https://github.com/mkelly9513/OV-Project-One/releases/tag/v1.1.3 (all bulk data analysis), single cell analysis can be found at https://github.com/RegnerM2015/scOVAR_SE_Screen, and Hi-C data analysis can be found at https://github.com/EricSDavis.

Bulk Data Analysis:
Python 3.6.5
R 4.0.0
Intervene 0.6.5
FastQC v0.11.7
bbduk.sh 38.46
STAR 2.6.0a
Samtools 1.9
htseq 0.11.2 - gencode v29
RSeQC 3.0.0
MultiQC 1.9
DESEQ2 1.30.1
SVA 3.3.0
Screen_Preprocessing.R (custom)
OVCAR3 _Screen_Analysis_ with_Plotting_LFC_ Comparison.ipynb (custom)
DESEQ2_RNA_Plotting_CRISPRi_Analysis_Revised.Rmd (custom)
DESEQ2_2021Reps_RNA_SVA_Plotting_ V2.Rmd (custom)

Split_ Genome_into_ windows.ipynb (custom)
OVLP _CNV _Whole_Genome.py (custom)
Combine_CNV _Chr _Files.ipynb (custom)
SEOVLP_CNV.py (custom)
CNV_eQTL.R (custom)
OVCAR_CNV_Comparison_Final.R (custom)
CNV_KM_Plots.R (custom)
Bedtools v2.25.0
fastq-dump 2.9.0
Trimmomatic 0.38
Picard MarkDuplicates 2.11.0
MACS2 2.2.6
bedGraphToBigWig v4
ROSE 0.1 (Python 2.7)
Deeptools 3.1.0
Bowtie2 2.3.5.1
CellProlifer4.2.1

Hi-C:
dietJuicer - https://github.com/EricSDavis/dietJuicer
bwa v0.7.17
R 4.1.0 - GenomicRanges (1.45.0), data.table (1.14.2), Homo.sapiens (1.3.1), InteractionSet (1.21.1), plyranges (1.13.1), ggplot2 (3.3.5), ggrepel (0.9.1)93. Example regions were visualized with the plotgardener (1.0.3).

Single-Cell:
Seurat 3.2
DoubletDecon 1.1.5
DoubletFinder 2.0.3
ArchR 0.9.3
ovar_3BAE2L_RNA-4.R (custom)
ovar_3E5CFL_RNA-4.R (custom)
ovar_HGSOC_RNA.R (custom)
HGSOC_ATAC.R (custom)
HGSOC_samples_SE_analysis.R (custom)
Motif_Analysis_SE60_SE14.sh (custom)
FIMO_TF_rank.R (custom)

H3K9me3 ChIP-seq performed by Active Motif:
bcl2fastq2 (v2.20)
bwa (v0.7.12)
Samtools (v0.1.19)
BEDtools (v2.25.0)
MACS2 (v2.1.0)
SICER (v1.1)
wigToBigWig (v4)

For manuscripts utilizing custom algorithms or software that are central to the research but not yet described in published literature, software must be made available to editors and reviewers. We strongly encourage code deposition in a community repository (e.g. GitHub). See the Nature Portfolio guidelines for submitting code & software for further information.

Data

Policy information about availability of data

All manuscripts must include a data availability statement. This statement should provide the following information, where applicable:

- Accession codes, unique identifiers, or web links for publicly available datasets
- A description of any restrictions on data availability
- For clinical datasets or third party data, please ensure that the statement adheres to our policy

Data generated in this study (CRISPRi/CRISPR KO RNA-seq, Hi-C, and H3K9me3 ChIP-Seq) have been uploaded and are publicly available in the Gene Expression Omnibus (https://www.ncbi.nlm.nih.gov/geo/) database under the accession number GSE174259 [https://www.ncbi.nlm.nih.gov/geo/query/acc.cgi?acc=GSE174259].
The single cell genomics data were downloaded from GEO accession number GSE17368243 [https://www.ncbi.nlm.nih.gov/geo/query/acc.cgi?acc=GSE173682]. The H3K27ac ChIP-seq and BRD4 ChIP-seq were downloaded from GEO accessions GSE10140828 [https://www.ncbi.nlm.nih.gov/geo/query/acc.cgi?acc=GSE101408] and GSE7756828 [https://www.ncbi.nlm.nih.gov/geo/query/acc.cgi?acc=GSE77568], respectively. All FTSEC data was downloaded from GEO accession GSE6810438 [https://www.ncbi.nlm.nih.gov/geo/query/acc.cgi?acc=GSE68104]. TCGA expression and copy-number data10 (RNA-seq and CNV data) was downloaded using the TCGA repository Firebrowse (http://firebrowse.org/) and survival data was attained from the supplement of the TCGA clinical paper44. All ENCODE data was downloaded from the Screen database37 (https://screen.encodeproject.org/#).
Kaplan Meier plots for gene set survival analysis were created with the publicly available KM Plot tool34,35 (https://kmplot.com/analysis/). Patient data from Figure 1b and Figure 1c are publicly available through CBioPortal6,31 (https://bit.ly/3QY91sa). The bar chart from Figure 1b can be found under Cancer Types Summary. The boxplot from Figure 1c can be generated under Plots by plotting TCGA PanCanAtlas Cancer Type Acronym vs. mRNA Expression, RSEM (Batch normalized from Illumina HiSeq_RNASeqV2) (log2(value+1)) and sorting the categories by median. For both Figure 1b and Figure 1c, the 16 highest altered/expressed TCGA cancer types are presented.
The remaining data are available within the article, Supplementary Information, or Source Data file.

Field-specific reporting

Please select the one below that is the best fit for your research. If you are not sure, read the appropriate sections before making your selection.

☒ Life sciences ☐ Behavioural & social sciences ☐ Ecological, evolutionary & environmental sciences

For a reference copy of the document with all sections, see nature.com/documents/nr-reporting-summary-flat.pdf

Life sciences study design

All studies must disclose on these points even when the disclosure is negative.

Sample size

No statistical approaches were used to predetermine sample size. Instead, we adopted industry standards and validated all experiment with robust comparisons across independent samples. Individual experimental designs are described below with explanations of sample size.
SE ChIP-Seq: There was a single replicate of H3K27ac and a single replicate of BRD4 data publicly available in OVCAR3. Since we were looking for co-localization of these independently generated signals and only needed general locations of super-enhancers, we felt confident this was sufficient for identifying regions of interest.
H3K9me3 ChIP-Seq: There were 2 independent replicates of scramble (non-targeting) sgRNA and 2 independent replicates of SE60 CRISPRi sgRNA H3K9me3 ChIP-Seq data generated with drosophila spike in and input made from all 4 samples. The spike in chromatin allowed for accurate quantification of signal and adjustment for technical noise across our 2 replicates, and replicates ensured statistically reliability with DESEQ2, an industry standard.
TCGA: We used RNA-Seq and copy number data from 300 patients for the CNeQTL analysis; for the CNV comparisons we used ~600 samples.
OVCAR3 CRISPRi Screen: As this was an exploratory analysis. Every sample was a single replicate, with 4 negative and 6 positive controls being utilized to assess success (86 experimental samples, 4 negatives, 6 positive controls). As all 96 samples are OVCAR3 cells treated with various sgRNas,, they were used collectively to establish the background expression of each gene (96 replicates). Data from all negative control samples was then used to establish an eFDR threshold value for each sample, following guidelines from the CMAP project.
CRISPR KO: In the CRISPR KO experiments, we had three control samples (wild type OVCAR3 cells) and four experimental samples for each super-enhancer investigated. For each super-enhancer, three independent knockout clones were used as biological replicates, one of the clones having two RNA-seq replicates, giving us a total of 4 KO replicates. 3 biological clones were used to ensure reproducibility and detection of true KO targets.
CRISPRi RNA-seq: There were two non-targeting (scramble) controls and 2 CRISPRi targeted replicates per SE (2 SEs) which allowed for statistically relevant detection of DEGs via DESEQ2.
Hi-C: We had 4 replicates of Hi-C data eventually merged into a single file for processing, following a modified Juicer Pipeline. We consulted with Hi-C expert Doug Phansteil for the experimental design (http://phanstiel-lab.med.unc.edu/).
Single Cell: We had tumors from two HGSOC patients which were used to generate single cell RNA-Seq and ATAC-Seq libraries. We had two sets of scRNA-Seq data and two sets of scATAC-Seq data.

Data exclusions

ChIP-Seq: No data was excluded in either study
TCGA: Samples without matching RNA-Seq and copy number were excluded from the CNeQTL analysis as this requires linked variation and expression data. All tumor samples were used for the copy number analysis.
CRISPRi Screen: Samples SE61 and SE64 were removed from the rank based analysis due to their outlier activity after VST normalization and PC investigation. Batch correction using SVA was unable to correct for technical issues present in these samples. All other samples clustered well, regardless of technical factors such as batch and day after removal of these samples.
CRISPR-KO/CRISPRi: All samples were kept for analysis since WT replicates and Experimental Replicates showed good correlation. The same is true of the CRISPRi followed by RNA-Seq.
Single Cell: No samples were excluded. Within samples, cells were kept only if meeting the following criteria; log(UMI counts) (>2 MADs, low end), log(number of genes expressed) (>2 MADs, low end) and log(percent mitochondrial read count +1) (>2 MADs, high end), followed by doublet detection.
H3K9me3 ChIP: No samples excluded
Hi-C: No samples excluded

Replication

ChIP-Seq: Independent data sets originating from two different labs (one for H3K27ac and one for BRD4) were used to determine final regions of interest; locations "identified" due to noise in any individual sample would not have been retained.
H3K9me3 ChIP-Seq: We generated two replicates of scramble (non-targeting) guide cells as controls and two replicates of SE60 targeted guide cells as our experimental condition. Drosophila chromatin spike-in was used to allow for extremely accurate quantification and input was generated from all samples to allow for normalization.
TCGA:There are hundreds of samples in the TCGA data sets we used; additionally we ran all analyses multiple times in order to ensure they could be replicated. We also made use of bootstrapping and permutation testing to ensure that results found with this data were reliable and robust. In addition when performing analysis such as the CNVeQTL analysis we calculated empirical false discovery rates from an null condition.
CRISPRi Screen:As there were only single replicates of each condition we decided to use all 96 samples to build and overall genomic background for gene expression. Additionally change in rank was utilized rather than gene expression to account for the unknown variation in the data. The negative control samples were used collectively to determine the "null behavior" of genes under this model and an empirical FDR of 0.1% was used to filter out false positive results.
CRISPR-KO:We ensured that our negative control replicates were closely correlated as were the experimental replicates per condition. Additionally we used three biologically independent CRISPR-KO's (with one of those KOs having a true replicate) as replicates to ensure that any results seen were not specific to a given knockout. Genes with additional lines of evidence linking them to these super-enhancers (genes predicted through independent CNVeQTL analysis, CRISPRi Screen, CRISPRi RNA-Seq, or Hi-C) or high significance values were the focus of the results. Proliferation and migration assays were repeated at least 3 times in order to derive significance values, these replicates strongly agreed with one another.
CRISPR-i: We ensured that our negative control replicates were closely correlated as were the experimental replicates per condition.

Additionally we used replicates to ensure that any results seen were reliable. Genes with additional lines of evidence linking them to these super-enhancers (genes predicted through independent CNVeQTL analysis, CRISPRi Screen, CRISPR-KO RNA-Seq) or high significance values were the focus of the results.
Hi-C: Four replicates of data were merged into a single analysis file following a modified JUICER pipeline (https://github.com/EricSDavis/dietJuicer) and the consultation of an expert Hi-C lab (http://phanstiel-lab.med.unc.edu/)

Randomization

TCGA: Permutation testing and bootstrapping were used to assess where randomized data agree with one another.
CRISPRi: The samples were randomly assigned to different wells on the the well plate; with assurance that all controls would be spread across the plate. There was no bias to assignment of well position outside of ensuring a spreading of the controls.
CRISPR-KO: We ensured that each condition (e.g one control and one KO for each SE) had libraries prepared on the same day and sequenced at the same time. We also utilized batch correction tools when performing differential expression analysis.
CRISPRi RNA-Seq: We ensured that each condition (e.g one control and one SE-targeting) had samples prepared on the same day, libraries created on the same day, and sequenced at the same time. We also investigated the need for batch correction tools when performing differential expression analysis and determined they would not be necessary.
H3K9me3 ChIP-Seq: We ensured that each condition (e.g one control and one SE60 targeted region group) had samples prepared on the same day, libraries created on the same day, and sequenced at the same time

Blinding

Blinding was used for microscopic imaging of cell migration assay transwell inserts. For the genomics analysis, blinding was not used and was it necessary for data collection or analysis because we took steps within the experimental design to limit biases and ensured that we always had replicated results, often using multiple methods, in order to circumvent issues arising from bias.

Reporting for specific materials, systems and methods

We require information from authors about some types of materials, experimental systems and methods used in many studies. Here, indicate whether each material, system or method listed is relevant to your study. If you are not sure if a list item applies to your research, read the appropriate section before selecting a response.

Materials & experimental systems

n/a	Involved in the study
☐	☒ Antibodies
☐	☒ Eukaryotic cell lines
☒	☐ Palaeontology and archaeology
☒	☐ Animals and other organisms
☒	☐ Human research participants
☒	☐ Clinical data
☒	☐ Dual use research of concern

Methods

n/a	Involved in the study
☐	☒ ChIP-seq
☒	☐ Flow cytometry
☒	☐ MRI-based neuroimaging

Antibodies

Antibodies used

anti-β-tubulin (Abcam, cat# ab6046) - used at 1:5000
anti-Cas9 (7A9-3A3) (Santa Cruz, cat# sc-517386) - used at 1:1500
H3K9me (Active Motif, Cat# 39161, Lot# 30220003) - 5ug per ChIP reaction (done by Active Motif)

Validation

All antibodies are commercially available and were used per the manufacturers' instructions and validations.

Eukaryotic cell lines

Policy information about cell lines

Cell line source(s)

NIH-OVCAR3 from ATCC
HEK-293T from ATCC

Authentication

The NIH-OVCAR3 cell line was authenticated using the ATCC Cell Line Authentication Service via STR profiling.

Mycoplasma contamination

The cell lines used in this study were tested and free of mycoplasma contamination.

Commonly misidentified lines
(See ICLAC register)

n/a

ChIP-seq

Data deposition

☒ Confirm that both raw and final processed data have been deposited in a public database such as GEO.

☒ Confirm that you have deposited or provided access to graph files (e.g. BED files) for the called peaks.

Data access links
May remain private before publication.

https://www.ncbi.nlm.nih.gov/geo/query/acc.cgi?acc=GSE174259

Files in database submission

GSE195676_RAW.tar
GSM5847954_0_0AY6_01LQUNC_Pooled_Input_hg19_i44_uniqnorm_signal.bw
GSM5847954_0_0AY6_01LQUNC_Pooled_Input_hg19_i44_uniqnorm_signal.bw
GSM5847956_2_0AXV_01LQUNC_SCR-2_H3K9me3_hg19_i96_dmnorm_signal.bw
GSM5847957_3_0AXW_01LQUNC_SE60-1_H3K9me3_hg19_i40_dmnorm_signal.bw
GSM5847958_4_0AXX_01LQUNC_SE60-2_H3K9me3_hg19_i43_dmnorm_signal.bw

Genome browser session
(e.g. UCSC)

No longer applicable.

Methodology

Replicates

We generated two replicates of scramble (non-targeting) guide cells as controls and two replicates of SE60 targeing guide cells as our experimental condition. Drosophila chromatin spike-in was used to allow for extremely accurate quantification and input was generated from all samples to allow for normalization.

ChIP-seq library generation and analysis was performed by Active Motif.

Sequencing depth

75bp single-end.

Total number of reads:
SCR-1 - 32,031,417
SCR-2 - 26,878,943
SE60-1 - 41,608,205
SE60-2 - 44,821,777
Input - 37,705,567

Uniquely mapped reads:
SCR-1 - 21,049,820
SCR-2 - 17,644,381
SE60-1 - 26,750,708
SE60-2 - 29,217,557
Input - 30,338,563

Antibodies

H3K9me (Active Motif, Cat# 39161, Lot# 30220003)

Peak calling parameters

Done by Active Motif. Information provided from Active Motif:
The generic term “Interval” is used to describe genomic regions with local enrichments in tag numbers. Intervals are defined by the chromosome number and a start and end coordinate. The peak caller used at Active Motif for this project was SICER89. This method was used to detect significant enrichments in the ChIP/IP data file when compared to the Input data file or relative to neighboring background regions.

Data quality

SICER cutoff is FDR 1e-10 with gap parameter of 600 bp.
Number of peaks:
SCR-1 - 31,750
SCR-2 - 33,510
SE60-1 - 36,871
SE60-2 - 30,431

Software

bcl2fastq2 (v2.20)
bwa (v0.7.12)
Samtools (v0.1.19)
BEDtools (v2.25.0)
MACS2 (v2.1.0)
SICER (v1.1)
wigToBigWig (v4)
